# Strategies to Improve School Meal Consumption: A Systematic Review

**DOI:** 10.3390/nu13103520

**Published:** 2021-10-07

**Authors:** Juliana F. W. Cohen, Amelie A. Hecht, Erin R. Hager, Lindsey Turner, Kara Burkholder, Marlene B. Schwartz

**Affiliations:** 1 Department of Public Health and Nutrition, Merrimack College, 315 Turnpike Street, North Andover, MA 01845, USA; 2 Department of Nutrition, Harvard T.H. Chan School of Public Health, 677 Huntington Ave., Boston, MA 02115, USA; 3 Institute for Research on Poverty, University of Wisconsin-Madison, 1180 Observatory Drive, Madison, WI 53706, USA; aahecht2@wisc.edu; 4 Departments of Pediatrics and Epidemiology & Public Health, University of Maryland School of Medicine, Baltimore, MD 21201, USA; ehager@som.umaryland.edu; 5 College of Education, Boise State University, 1910 University Drive, Boise, ID 83725, USA; lindseyturner1@boisestate.edu; 6 College of Liberal Arts and Science, University of Connecticut, Storrs, CT 06269, USA; kara.burkholder@uconn.edu; 7 Rudd Center for Food Policy and Obesity, Department of Human Development and Family Sciences, University of Connecticut, 1 Constitution Plaza, Suite 600, Hartford, CT 06103, USA; marlene.schwartz@uconn.edu

**Keywords:** school meals, nutrition, breakfast, lunch, choice architecture, nutrition education, taste tests, policies, recess, choices, palatability, pre-sliced, competitive foods

## Abstract

School meals can play an integral role in improving children’s diets and addressing health disparities. Initiatives and policies to increase consumption have the potential to ensure students benefit from the healthy school foods available. This systematic review evaluates studies examining initiatives, interventions, and policies to increase school meal consumption. Following PRISMA guidelines, this review was conducted using four databases and resulted in a total of 96 studies. The research evidence supports the following strategies to increase school meal consumption: (1) offering students more menu choices; (2) adapting recipes to improve the palatability and/or cultural appropriateness of foods; (3) providing pre-sliced fruits; (4) rewarding students who try fruits and vegetables; (5) enabling students to have sufficient time to eat with longer (~30 min) lunch periods; (6) having recess before lunch; and (7) limiting students’ access to competitive foods during the school day. Research findings were mixed when examining the impact of nutrition education and/or offering taste tests to students, although multiple benefits for nutrition education outside the cafeteria were documented. There is some evidence that choice architecture (i.e., “Smarter Lunchroom”) techniques increase the proportion of students who select targeted meal components; however, there is not evidence that these techniques alone increase consumption. There were limited studies of the impact of increasing portion sizes; serving vegetables before other meal components; and strengthening local district and/or school wellness policies, suggesting that further research is necessary. Additionally, longer-term studies are needed to understand the impact of policies that limit students’ access to flavored milk. Several studies found increases in students’ meal consumption following the Healthy Hunger-Free Kids Act (HHFKA) and concerns regarding an increase in food waste following the HHFKA were not supported. Overall, there are a range of effective strategies to increase school meal consumption that can be implemented by schools, districts, and policymakers at the local, state, and federal levels (PROSPERO registration: CRD42021244688).

## 1. Introduction

In the United States, approximately 95% of public and non-profit private elementary, middle, and high schools participate in the school meal programs administered by the United States Department of Agriculture (USDA) [1,2]. Both the National School Lunch Program (NSLP) and School Breakfast Program (SBP) provide children and adolescents with healthy, low-cost meals throughout the school year. Prior to COVID-19, approximately 30 million children received school lunches daily, and roughly three-quarters of school lunch participants come from low-income households, with many relying on school meals for up to half of their daily energy intake [3,4]. Schools are therefore uniquely positioned to promote healthy eating among children.

In 2010, the Healthy, Hunger-Free Kids Act (HHFKA) required the USDA to update many of its regulations for federal school nutrition programs to reflect the concurrent Dietary Guidelines for Americans [5]. Updates to the NSLP included requirements for more whole grain-rich foods; greater portion sizes for fruits and vegetables; a greater variety of vegetables offered throughout the week; limits on total calories and sodium; and the removal of *trans* fats. Nutrition standards were also strengthened for “competitive” foods, defined as snacks and beverages sold during school hours, but outside of the school meal programs (i.e., in vending machines, à la carte, snack bars, school stores, and fundraisers) [6]. Lastly, schools were required to update their local school wellness policies to promote healthier school environments [7]. 

**Figure 1:**
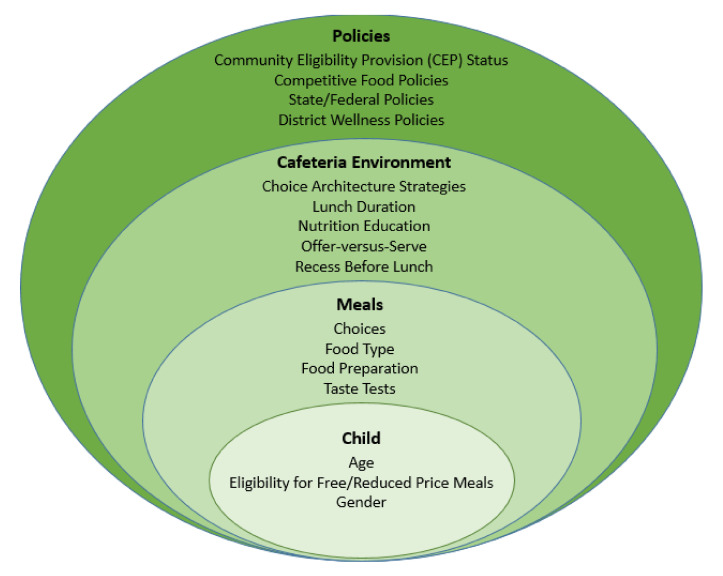
Social Ecological Framework of the Influences on School Meal Consumption (adapted from Graziose and Ang 2018 [23]).

The evidence regarding school meal consumption suggests that rates are influenced by multiple intersecting factors, including at the individual, meal, cafeteria environment, and policy levels (Figure 1). At the *individual level*, consumption varies by student age, gender, and eligibility for free or reduced-priced meals (based on family income), as well as factors such as preferences and prior exposure to foods [8,9,10]. 

At the *meal level*, consumption consistently varies by food type (i.e., the federal meal components: meat/meat alternatives; grains; fruits; vegetables; and milk) with students typically consuming lower quantities of fruits and vegetables compared with the other components [11,12,13]. Although schools are not allowed to deviate from the federal meal pattern requirements, they may modify other meal service factors, such as offering students multiple choices of each required meal component; providing taste tests; and changing how the food is prepared (e.g., improving palatability/cultural appropriateness and pre-slicing fruits). Providing multiple food items from which students can choose increases the likelihood that one option will be appealing to a student. Increasing choices for students can occur on the lunch line (e.g., student can select between two fruit options) or through salad bars, which can hold a variety of produce. Palatability is also important to address as it is a primary determinant of food consumption, and familiarity with a food is strongly correlated with preferences [14,15]. Therefore, focusing on culturally appropriate school meals that emphasize familiar flavors and taste may be a key component to ensuring high meal consumption rates. Interventions that involve collaborating with professional chefs or seasoning foods (both prior to serving or at “flavor stations” for students to add their own spices) have the potential to enhance the palatability and cultural appropriateness of school meals. Additonally, pre-slicing fruits may impact consumption because whole fruits, such as apples and oranges, may be challenging to consume if students have a limited amount of time to eat, perceive the fruit as messy, or have difficulty holding and biting the fruit (particularly among younger children or those with braces). Lastly, taste tests may provide the opportunity for repeated exposures to new foods, as well as a chance for students to provide feedback to cafeteria staff, thus influencing the options on the school menu [16].

At the *cafeteria environment level, modifications* can potentially influence consumption via choice architecture strategies to nudge students towards healthier options; changing the length of the lunch periods to ensure students have sufficient time to eat; providing nutrition education; and scheduling recess before lunch. Choice architecture, also known as behavioral economics, uses strategies to “nudge” people towards the healthier options available in food environments [17]. Choice architecture strategies can include rewarding students for selecting and/or consuming meal components; implementing traditional Smarter Lunchroom techniques (e.g., visually appealing displays, verbal prompts, and creative names for foods); altering the portion size of foods; and modifying the timing of different meal components (e.g., making vegetables available before fruit). Ensuring a sufficient amount of time to eat is also important, but school lunch durations vary greatly and typically range from 15–45 min [18]. Additionally, the time scheduled for the lunch period includes time needed to walk to the cafeteria, wait in line for lunch, and clean up, resulting in substantially less time for students to actually eat their meals [19]. Nutrition education in schools (which can include a range of activities, such as traditional instruction regarding nutrients and food groups, garden-based farm-to-school lessons, and skill-based cooking classes) can also be important to provide children with the skills and knowledge necessary to make informed and healthy food choices [20]. Additionally, recess traditionally occurs after school lunch in the United States, but reversing the order may impact school meal consumption. It has been hypothesized that recess before lunch may enable students to expend more energy and thus be hungrier at lunch time, as well as prevent students from rushing through lunch for more play time at recess [21]. Recess before lunch may also push back the start time of lunch to more traditional lunch hours when students may be hungrier. Of note, implementing offer-versus-serve (i.e., not requiring students to select all the meal components offered) is a commonly recommended strategy to reduce waste, but it cannot increase consumption because students are accessing less food [22]. 

Lastly, at the *policy level*, regulations can be implemented at the local, state, and federal level to support increases in school meal consumption. These can include strong district wellness policies; access to universal free school meals (e.g., participation in the Community Eligibility Provision (CEP) in schools where at least 40% of students come from lower-income households); restricting access to competitive foods; and strengthening meal nutrition standards. Local polices (including district wellness policies) have the flexibility to go beyond state or federal regulations, and also have the benefit of supporting the unique needs of individual schools and districts. There are many different interventions that can be written into policies, including serving breakfast in the classroom, which may increase breakfast consumption because students are given more time to eat than they might otherwise have between when they arrive at school and classes start. Strong polies for competitive foods (i.e., foods and beverages sold outside of meals that may “compete” with school meals) may be important as they potentially impact both participation in the NSLP (via replacement of “competitive foods” that are purchased instead of school meals) and consumption if a student has both a snack and a school lunch. 

Additionally, it is important to note the nuanced similarities and differences between the variables of school meal selection, consumption, and waste. Selection is a necessary pre-requisite for consumption. Importantly, when the total number of students selecting a meal component increases, there may be benefits at the population level even without increases in the proportion consumed for each child who selected the food. For example, if there is an increase in the number of students selecting fruit, and the average level of fruit consumption remains at 50% per child who selected fruit, a greater number of students are eating half a fruit at lunch. Therefore, an increase in selection can result in overall increases in consumption for the student population as a whole, even when the proportion of a meal component consumed among children who selected the food remains the same. Waste is the opposite of consumption when examining the percent consumed of an individual meal component. However, waste may not be the opposite of consumption when examining these variables if multiple servings of a meal component can be taken by students (e.g., if a student selects two pieces of fruit and at least one is partially consumed, both consumption [measured in servings] and waste can increase). Additionally, if selection increases, and the proportion consumed per serving selected remains the same, both overall consumption and overall (aggregate) waste at the population level will increase. For these reasons, it is important to address changes in selection when comparing consumption and waste.

The problem of food waste in the NSLP has been documented for decades, and the consistently low levels of consumption for various food types, especially vegetables and fruits, remains a problem [24,25,26,27]. However, a scoping review of research examining factors that impact school meal consumption has not been conducted. Several systematic reviews have examined techniques to nudge students towards healthier options (i.e., choice architecture), but many of the included studies only measured selection [28,29,30,31,32,33,34]. Other recent school-based reviews have been limited in scope, such as examining only fruits and vegetables [23,35,36]. A better understanding of initiatives, interventions, and policies that can improve school meal consumption more broadly is needed to help inform school food service programs and policies at the district, state, and federal levels. Therefore, the aim of this study was to systematically review the evidence regarding the impact of various strategies to improve school meal consumption. 

## 2. Materials and Methods

The review was conducted in accordance with the Preferred Reporting Items for Systematic Reviews and Meta-Analysis (PRISMA) guidelines [37]. This literature review was registered with the PROSPERO International Prospective Register of Systematic Review before data extraction (protocol registration number: CRD42021244688) [38]. 

### 2.1. Data Sources and Search Strategies

Articles were retrieved from four electronic databases: PubMed, Academic Search Ultimate, Education Resources Information Center (ERIC), and Thomson Reuters’ Web of Science. The search strategy used varying combinations of the following keywords: school AND (meal OR breakfast OR lunch) AND (intake OR eat * OR consum * OR waste) AND (atmosphere OR behavioral economics OR breakfast in the classroom OR chef OR choice OR competitive food OR cultur * OR default OR duration OR engag * OR environment OR label * OR length OR marketing OR menu OR minutes OR nudg * OR nutrition education OR offer OR palatab * OR placement OR policy OR promotion OR repeat * OR slic * OR smarter lunchroom OR taste OR wellness). Articles in English published since the start of the literature through May 2021 were reviewed. Manual searches of the articles’ reference lists, as well as a review of all articles citing the resultant literature (using Google Scholar), were conducted to identify other potentially relevant studies.

### 2.2. Study Selection

Eligible studies were quantitative research articles evaluating interventions, initiatives, and policies to influence school meal consumption. Our inclusion criteria were English, peer-reviewed publications or official government reports conducted in the United States within elementary, middle, and/or high schools participating in the USDA’s SBP and/or NSLP during the academic year. The following types of articles were excluded: non-English articles; qualitative research; articles that did not examine school breakfast or lunch (e.g., only snacks or afterschool programs); studies conducted in private and/or charter schools not participating in the SBP or NSLP; initiatives that occurred outside of the school year (i.e., summer vacations); studies conducted in locations outside the United States; studies that did not quantify the amount of the entire school meal or school meal component consumed (i.e., assessing only school meal selection, binary (yes/no) estimates of consumption, estimates of only the nutrients consumed at lunch, and/or food frequency questionnaires or 24-hour recalls assessing only overall diets); and articles that did not examine initiatives, interventions, or policies (e.g., differences in consumption by age, gender, etc.). Additionally, studies examining universal free school meals and/or the Community Eligibility Provision were excluded as this was recently evaluated in a systematic review [39]. Titles and abstracts were screened by two independent reviewers, and full texts were screened by the lead author (JFWC) based on the eligibility criteria. The research team reviewed articles with unclear eligibility. The heterogeneity in the methods used to assess consumption and the variability in study designs precluded a meta-analysis; therefore, the results were synthesized in a narrative review. 

### 2.3. Quality Assessment

Study quality and biases were assessed using the previously adapted Newcastle–Ottawa Scales (NOS) for cross-sectional and cohort studies, which are frequently used to assess non-randomized, community-based research [39,40,41,42]. The studies were evaluated by two co-authors based on selection, comparability, and outcome and categorized as: low risk of bias (≥7 points), high risk of bias (4–6 points), and very high risk of bias (0–3 points) [43]. Supplementary Materials Table S1 presents the quality assessments of the included studies. 

## 3. Results

**Figure 2:**
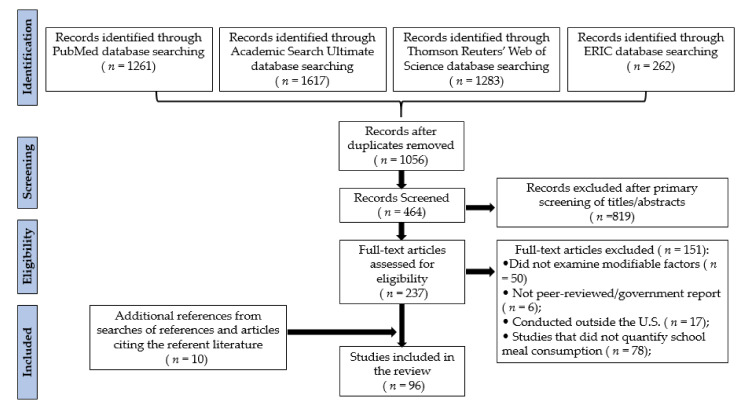
Flow chart for systematic review.

The initial search of the four databases (PubMed, Academic Search Ultimate, ERIC, and Thomson Reuters’ Web of Science) identified 4423 articles. A total of 3367 duplicate records were removed, and primary screening excluded an additional 819 articles. The full text of the remaining 237 publications were examined in detail, and 151 studies were excluded. The primary reasons for exclusion were based on the study objectives (i.e., did not examine initiatives, interventions, or policies related to school meal consumption); the outcomes assessed (i.e., did not quantify the amount of school meals consumed or wasted); publication type (i.e., grey literature); and the study location (i.e., outside the United States). Ten additional articles were identified from the reference lists of the obtained articles or publications citing those studies, resulting in a total of 96 articles included in the review (Figure 2). The quality scores for the articles included in the review ranged from 2 (very high risk of bias) to 10 (low risk of bias) (Supplementary Materials Table S1). Approximately a third of the included studies (*n* = 33) were classified as having a low risk of bias, over half of the articles (*n* = 55) had a high risk, and the remaining articles (*n* = 8) had a very high risk. The studies are presented by the different initiatives, interventions, and policies. Many studies included several components and are thus described in multiple sections.

### 3.1. Initiatives and Interventions Related to School Meals 

#### 3.1.1. Food Choices

Of the 12 peer-reviewed publications that examined offering more options from which students could choose, the majority (*n* =8 studies) found significant positive associations with consumption; one found no association; one had mixed findings; and two found a decrease in consumption (Table 1). Three studies were considered to have a low risk of bias, and of those, two found a positive association with consumption. 

In a study using visual estimation in eight elementary and four middle schools in Houston, Texas, Cullen and colleagues found that when students had the ability to select from more fruit and vegetable options, elementary students consumed more vegetables (0.14 vs. 0.10 cups; *p* < 0.01), and middle school students consumed more whole fruits (0.19 vs. 0.10 cups; *p* < 0.001) and vegetables (0.17 vs. 0.10 cups; *p* < 0.01) [44]. Additionally, this study found significant increases in selection of these meal components. Using similar consumption measures, Just et al. examined the impact of increasing the number of fruit and vegetable options at lunch in 22 elementary schools and found that each additional option available increased the number of students who ate at least one serving of these meal components by 3.3 percentage points (*p* < 0.05) [45]. Hakim and associates also observed increases in both fruit and vegetable consumption by roughly 15% (*p* < 0.01) using weighed plate waste and visual estimation in one K-8 school when students were given a choice among three fruit or vegetable options [46]. Using the same consumption measure, Young and colleagues also found an increase in fruit and cooked vegetable consumption when students in one middle school had access to different fruits and vegetables every day of the week (this intervention also included exposure to increased health and physical education) [47]. In another multi-component study in 26 elementary schools in Minnesota, Perry et al. used visual estimation to examine the impact of offering multiple fruit and vegetable choices and found a 0.15 serving increase in combined fruit and vegetable consumption (*p* = 0.02). This intervention also included pre-slicing fruit and choice architecture techniques (e.g., visually appealing displays, verbal prompts, and rewards) [48]. A similar multi-component study by Greene and associates in seven middle schools in New York assessed consumption with visual estimation and found that multiple fruit and vegetable options (again, in addition to pre-slicing fruit and choice architecture techniques [e.g., attractive bowls and descriptive names]), was associated with a 23% increase in fruit consumption (*p* < 0.017), as well as a significant increase in selection. In the Green study, however, there was no impact on vegetable (or milk) selection or consumption [49]. While another study by Liquori et al. did find an increase in vegetable consumption (also measured using visual estimation) in two New York City elementary schools, this multi-component study only found an effect when students had a greater number of choices and cooking lessons, but not when choices were combined with traditional nutrition education compared with students in control schools [50]. Additionally, Ang and colleagues found no impact on fruit consumption using visual estimation when students in New York City elementary schools had access to multiple fruit options [51].

The results among the four studies examining salad bars were mixed. In a study in two California elementary schools that included both salad bars and nutrition education (including gardening and cooking demonstrations), Taylor and colleagues found a significant increase in vegetable consumption measured using digital imagery: intake went from 0.09 cups pre-intervention to 0.15 cups post-intervention while consumption declined slightly in the control schools from 0.05 to 0.04 cups (*p* = 0.03) [52]. In contrast, Bean et al. found consumption decreased by 0.65 cups (*p* < 0.001) using digital imagery when students in two elementary schools in Virginia had access to a salad bar, although there was a significant increase in the number of fruits and vegetables selected (1.81 vs. 2.58; *p* < 0.001) [53]. Similar findings were observed in a cross-sectional study by Johnson et al. using 24-hour recalls among middle and high school students attending 21 schools in New Orleans, with less fruit consumption reported among students with a salad bar (and no impact on vegetable consumption) [54]. Interestingly, Adams et al. found that while salad bars alone did not impact the amount of fruits or vegetables that students ate, a greater number of options was associated with increased fruit and vegetable consumption using weighed plate waste in four elementary schools in San Diego, California [55]. 

#### 3.1.2. Food Preparation: Palatability and Cultural Appropriateness

Of the nine peer-reviewed publications that examined palatability, six found a positive association with school meal consumption; one found no association; one had inconsistent findings; and one found an inverse association (Table 1). Four studies had a low risk of bias; four had a high risk; and one had a very high risk. Of the studies with a low risk of bias, all found that enhanced palatability of school meals were positively associated with consumption.

Multiple studies have examined the impact of professional chefs in schools, and nearly all (four out of five) found positive associations with school meal consumption. In a pilot study conducted in four middle schools in Boston, Cohen and colleagues measured consumption using weighed plate waste; they found that students who received chef-enhanced meals consumed 0.36 more servings of vegetables compared with students in control schools (*p* = 0.01) [56]. Additionally, this study found a significant increase in whole grain selection, resulting in more students consuming whole grains. In a study among 14 elementary and middle schools in Massachusetts using similar methods, Cohen et al. found an increase in both fruit (0.34 vs. 0.51 cups; *p* < 0.05) and vegetable (0.14 vs. 0.30 cups; *p* < 0.05) consumption among students in schools with a professional chef compared to students in control schools (as well as increased selection of these meal components) [57]. These findings of increased fruit and vegetable consumption were replicated by Cohen et al. in a third study conducted in eight elementary and middle schools in Massachusetts after implementation of the HHFKA [58]. A study conducted by Zellner and associates in two elementary schools in Philadelphia examined chef-prepared meals (in addition to family-style service and an adult at the table as a role model) using visual estimation measurement and found that students consumed more of the targeted vegetables (sweet potato fries and cauliflower) [59]. Lastly, Just et al. conducted a study in one high school in New York with chef-enhanced pizza recipes (and taste tests) using visual estimation measurements; they found no impact on consumption, although increasing pizza consumption was challenging due to its very high baseline consumption rates [60]. However, an unintended consequence of this study was that more students received a side salad with the pizza, and vegetable consumption increased.

Other studies have used alternate strategies to enhance the palatability of the foods offered. D’Adamo and colleagues added spices and herbs to vegetable recipes and selected recipes based on taste tests with students. Using weighed plate waste, this study found the enhanced recipes were associated with an 18% increase in vegetable consumption (*p* < 0.001) [61]. Conversely, in another weighed plate waste study in 1 middle/high school in Pennsylvania, Fritts et al. found that when spices and herbs were added to vegetables, consumption decreased for several vegetables [62]. This study also found no impact on consumption of those vegetables with repeated exposures. Hamdi and associates used weighed plate waste in a study in three elementary schools in Illinois [63]. They evaluated a flavor station where students could add their own spices and seasoning to their lunches (in addition to taste tests and choice architecture techniques) and found inconsistent improvements with consumption with the components differing in effectiveness between the two participating schools, although the odds of vegetable selection were three times greater (95% CI, 1.3–6.5). [63]. Lastly, Bates et al. conducted a study in one middle and one high school in Utah where fruit smoothies were offered to students at breakfast; using visual estimation, they found offering smoothies was associated with a 0.45 serving increase in fruit consumption (*p* = 0.01) [64].

#### 3.1.3. Food Preparation: Pre-Slicing Fruits

Among the eight studies examining the impact of offering pre-sliced fruit, the majority (*n* = 6 studies) found a positive association with consumption, and two found no association (Table 1). Four studies had a low risk of bias and the majorty (*n* = 3 studies) found pre-sliced fruit was associated with increased consumption. 

In a study conducted by Smathers and colleagues in two elementary schools using aggregate plate waste, pre-slicing apples versus serving them whole was associated with increased consumption (2.48 times more apples by weight [*p* < 0.001]) [65]. Using similar consumption measures, McCool et al. also found that consumption increased when pre-sliced apples were offered in an elementary school, and when middle school students were given the choice of pre-sliced or whole apples (some students preferred pre-sliced while others preferred whole fruit) [66]. Increased consumption of pre-sliced apples was also observed by Wansink and associates in six middle schools using visual estimation, with the percent of students eating at least half of an apple increasing by 73% (*p* = 0.02) [67]. In the previously described multi-component studies by Ang et al. and Greene et al., student consumption also increased when fruit was pre-sliced [49,51]. Conversely, Swanson and colleagues examined both sliced apples and oranges in one elementary school in Kentucky, and found that orange (but not apple) consumption increased [68]. Lastly, two multi-component studies found no association between serving pre-sliced apples and fruit consumption: Thompson et al. [69] used weighed plate waste in two elementary schools in Minnesota and Quinn et al. [70] used visual estimation in 11 middle and high schools in Washington. Among the six studies that measured selection, five found increases in selection with pre-sliced fruits.

#### 3.1.4. Taste Tests

Taste tests were one of the strategies used in nine multi-component studies, and were the sole intervention in one study. Four studies found positive associations with consumption; two had mixed findings; and four found no association (although in two of the studies, taste tests were inconsistently available in the participating schools [Table 1]). Six studies had a low risk of bias, and most (4 out of 6 studies) found a positive association between taste tests and school meal consumption.

Mazzeo and colleagues examined the impact of taste tests (and choice architecture techniques [i.e., stickers as a reward for trying foods]) using visual estimation in two elementary schools and found they were associated with reduced fruit and vegetable waste by roughly 10% (39% waste in intervention schools vs. 52% in control schools [*p* < 0.05]) [71]. In a multi-component study also using visual estimation and conducted in six elementary schools in Utah, Morril et al. evaluated the impact of taste tests in addition to choice architecture techniques (e.g., a reward for eating fruits and vegetables) and found an increase in fruit and vegetable consumption [72]. Additionally, the previously mentioned multi-component study by Perry and colleagues found that taste tests combined with more fruit and vegetable choices and choice architecture techniques was associated with an increase in fruit and vegetable consumption [48]. Alaimo and colleagues examined taste tests and nutrition education using digital imagery in six elementary schools in Michigan and found increases in fruit consumption, but not in consumption of vegetables or other meal components [73]. Similarly, a government report examining taste tests and nutrition education in one elementary school in Oregon found increases in fruit consumption using weighed plate waste [74]. While no impact on vegetable or grain consumption were observed, this study found significant increases in their selection (as well as fruit selection). Another study with taste tests and nutrition education in 16 elementary schools in Little Rock, Arkansas was conducted by Blakeway et al. They found inconsistent results using aggregate plate waste; increases in consumption of whole wheat rolls and cottage cheese were observed in some (but not all) grades [75]. As previously noted, similarly inconsistent results were observed by Hamdi and colleagues in a study that evaluated taste tests in combination with a flavor station and choice architecture techniques [63]. No association was observed in three studies that involved taste tests and nutrition education in 28 elementary schools in Alabama by Reynolds and associates using visual estimation [76], and in multiple elementary schools in Wyoming by Bontranger Yoder and colleagues using digital imagery [77,78], although taste tests were not available in all the participating schools in the latter two studies. Similarly, no impact on consumption was observed in the study by Just and colleagues when examining taste tests and chef-enhanced pizza recipes [60]. 

### 3.2. Initiatives and Interventions Related to the Cafeteria Environment

#### 3.2.1. Choice Architecture

Of the 23 peer-reviewed publications that primarily examined choice architecture, roughly half (*n* =13 studies) found a positive association with school meal consumption; seven studies found no association; and three studies found an unexpected increase in food waste (Table 2). Among the five studies with a low risk of bias, four were positively associated with consumption while one found an increase in food waste. When examining the ten multi-component studies (e.g., including nutrition education, taste tests, choices, and pre-sliced fruit), half found a positive association and half found no association. Seven of these studies had a low risk of bias and the results were also inconsistent. Overall, these mixed findings may be due in part to the fact that there are many different choice architecture techniques and success may vary by the specific strategy implemented. 

##### Rewards

Eleven studies tested the impact of providing rewards for students within elementary schools. Among those that required students to eat the fruits and vegetables prior to receiving the reward, nearly all (9 out of 10) found a positive association with fruit and vegetable consumption. The one study that only required selection (but not consumption) for a reward found no association with consumption. Hendy and colleagues conducted a study using visual estimation in one elementary school in Pennsylvania and found that providing children with a small prize for eating at least 1/8 cup of fruits and vegetables at lunch was associated with an increase in consumption [79]. Using the same measure of consumption, the previously described study by Perry and colleagues found that rewards (i.e., frozen yogurt for the classroom), as well as taste tests and more fruit and vegetable choices, were associated with an increase in fruit and vegetable consumption [48]. Jones et al. used aggregate plate waste in one elementary school in Utah and found that teacher encouragement to eat more fruits and vegetables combined with rewards (medals and points displayed on a board) was associated with a 39% increase in fruit consumption (*p* < 0.01) on the days when fruit was targeted and a 33% increase (*p* < 0.05) in vegetable consumption on the days when vegetables were targeted. Notably, neither fruit nor vegetable consumption increased when the other meal component was targeted [80]. In a study using digital imagery measurement in one Utah elementary school, Wengreen and colleagues found an increase in fruit and vegetable consumption when students received a reward for trying those meal components; this intervention also included educational videos and letters read by teachers promoting fruits and vegetables [81]. Using similar consumption measures in one elementary school in Oregon, Machado and colleagues provided verbal prompts, adult role modeling, and rewarded students for consuming fruits and vegetables with a classroom party and t-shirts. They found that the proportion of students consuming all their fruits increased by 11% (*p* < 0.01) and the proportion eating all their vegetables increased by 8.7% (*p* < 0.01) [82]. Increases in fruit and vegetable consumption were also observed in the previously described multi-component study conducted by Morrill et al., and decreases in waste for fruits and vegetables were observed in the study by Mazzeo et al.; both studies included taste tests as well [71,72].

In two studies conducted by Hoffman et al. within four elementary schools in New England, there were promotional posters, verbal encouragement, and students received a small prize (e.g., stickers) for eating fruits and vegetables. Both studies found an increase in consumption of fruits and vegetables using weighed plate waste [83,84]. However, they found the results were not sustained after the intervention concluded [84]. In another study, Blom-Hoffman et al. used visual estimation in one elementary school and found no impact on vegetable consumption when examining the impact of nutrition education combined with sticker rewards and verbal praise [85]. Hudgens and associates used visual estimation measurement in one elementary school in Oregon and found that providing a reward for selecting (but not requiring student to taste) a meal with a fruit, vegetable, unflavored milk, and whole grain was not associated with an increase in consumption, although increases in selection of these components were observed [86]. 

##### Smarter Lunchroom Strategies

Sixteen studies examined the visual appeal of the cafeteria environment—such as attractive bowls, signage with creative names—as well as verbal prompts, and/or location of fruits/vegetables. Among these studies, only four found a positive association with consumption; three found an increase in food waste; eight found no association with consumption; and one found inconsistent results. 

In a study conducted by Gustafson et al., children in four elementary schools in Nebraska designed posters marketing vegetables that were then displayed over a salad bar. They used digital imagery to measure plate waste and observed an increase in vegetable consumption [87]. Adams et al. used weighed plate waste for a study in middle schools in Phoenix, Arizona and tested the impact of moving the location of the salad bars. They found that students consumed 4.82 times more fruits and vegetables when the salad bar was accessible from the serving line compared with when salad bars were located after students had left the lunch line [88]. In the previously described multi-component study by Greene et al., attractive bowls and descriptive names were combined with providing pre-sliced fruit and increased fruit and vegetable choices. They found an increase in fruit consumption, but no impact on vegetable or milk intake [49]. Hanks et al. found that an intervention that included using attractive bowls and descriptive names in combination with changing the placement of foods, providing verbal prompts, and creating healthy convenience lines was associated with an 18% increase in fruit consumption (*p* = 0.004) and 25% increase in vegetable consumption (*p* < 0.001) in two middle/high schools in NY using visual estimation [89].

Although the previous studies found impacts on student consumption, these findings were not replicated in other studies and many studies found null effects. Using consumption measures similar to Hanks et al. [75], Quinn and associates used Smarter Lunchroom techniques (and pre-sliced fruit) and found no impact on fruit and vegetable intake in 11 middle and high schools in Washington [70]. Similarly, Thompson et al. found no impact of these interventions (i.e., Smarter Lunchroom strategies and pre-sliced fruit) on intake in two elementary schools in Minnesota using weighed plate waste [69]. Additionally, Cohen et al. used weighed plate waste to assess similar choice architecture techniques, as well as more prominent white milk (compared with chocolate milk) in 14 elementary and middle schools in Massachusetts, and found that these strategies had no impact on the consumption of fruits, vegetables, or milk [57]. Goto and colleagues increased the quantity and prominence of white milk in three elementary schools in California, and found no association with milk consumption using weighed plate waste [90]. Hanks and colleagues examined the exposure of only a healthy convenience line in a high school in New York using weighed plate waste and also found no impact on healthier meal consumption [91]. Additionally, placing vegetables first on the lunch line had no impact on consumption in a study conducted by Ang and colleagues in 14 elementary schools in New York City using visual estimation [51]. Promotional signage, a more open lunch line, wall art, and comfortable seating options was associated with reduced consumption of fruits and grain and no impact on vegetable consumption after a year of exposure in a study conducted by Koch et al. in seven high schools in New York City using digital imagery [92]. In a study conducted by Blondin et al. in six elementary schools using weighed plate waste, verbal encouragement in the classroom was found to increase milk waste [93]. This study also found that milk consumption was adversely impacted by the presence of juice. Schwartz et al. also examined verbal prompts (on the lunch lune) in two elementary schools in Connecticut using visual observation and found that while there were no differences in individual-level consumption rates between intervention and control schools (~70% consumption in both schools), there were significant increases in selection, and thus 70% of children consumed a serving of fruit in the intervention schools compared with less than 40% in the control schools [94]. As previously mentioned, inconsistent results were observed by Hamdi and colleagues in a study that evaluated choice architecture (in addition to taste tests and a flavor station) [63], and another study by Wansink et al. using visual estimation in one high school with posters and student involvement in growing the salad greens was associated with a decrease in salad consumption [95]. Similarly, Reicks and colleagues found that including pictures of vegetables on lunch trays was associated with a decrease in carrot consumption (and no association with green beans) using aggregate plate waste in one elementary school in Minnesota [96]. Importantly, nearly all (*n* = 12) studies examining Smarter Lunchroom Strategies found increases in selection.

##### Portion Sizes and Providing Meal Components at Different Times

A small number of publications examined portion sizes (2 studies) and providing meal components at different times (3 studies). While all found positive associations with consumption, only one study had a low risk of bias. Among studies examining portion sizes, Ramsay et al. increased the portion size of chicken nuggets provided to kindergarteners in one school and found increased consumption (and selection) using weighed plate waste [97], although processed foods such as chicken nuggets tend to have high consumption rates more broadly in schools [98]. In another study examining portion sizes, Miller and colleagues increased the portion sizes for fruits and vegetables by 50% and found a corresponding increase in consumption by about 13-42 g using weighed plate waste in one elementary school in Minnesota [99]. In this study, selection of some components increased while other components decreased. Two other studies conducted in an elementary school in Minnesota found that offering vegetables (i.e., red peppers, baby carrots, and broccoli) first while students waited in the lunch line was associated with increases in consumption using visual estimation (Elsbernd et al.) [100] and aggregate plate waste (Redden et al.) [101]. However, only the study by Eslbernd et al. found increases in selection as well. Similarly, in one elementary school in Philadelphia, Zellner and colleagues found that when offering fruit was delayed until later in the meal, students consumed more kale compared with when fruit was served at the same time as the vegetable (and entrée and milk) using visual estimation [102]. 

#### 3.2.2. Nutrition Education

Of the 11 studies focused on nutrition education alone, or with small additional components, such as parent newsletters, slightly over half (*n* = 6 studies) found a positive association with school meal consumption; four found no association; and one found a decrease in consumption (Table 3). Five studies had a low risk of bias, and the majority (*n* = 4 studies) found positive associations between nutrition education and consumption. Among the 10 multi-component studies that combined nutrition education with other strategies (e.g., taste tests, more food choices, and choice architecture techniques), slightly fewer than half (*n* = 4 studies) found increases in consumption; four found no association; and two found mixed results. Among the three multi-component studies with a low risk of bias, only one found a positive association with consumption.

In one of the studies that found a positive association between nutrition education and consumption, Sharma et al. implemented a 16-week nutrition education program in three elementary schools in Texas. This intervention also included a parent component with recipes and demonstrations and fresh fruit from local pantries sent home with families. They used weighed plate waste and found a decrease in fruit and vegetable waste at lunch (β = -32.06; *p* < 0.001), and while no differences in selection were observed in the intervention schools, significant decreases in selection were observed in the control school [103]. In a study conducted in four elementary schools in Denver by Auld and colleagues, students were exposed to 16 nutrition lessons taught alternatively by teachers and special resources teachers [104]. This study examined food consumption using visual estimation and found that nutrition education was associated with an increase in fruit and vegetable consumption by 0.36 servings (*p* < 0.001). Auld et al. conducted another study using similar methodology in 10 elementary schools in Denver, but with 24 nutrition lessons taught exclusively by a special resources teacher and found a similar effect size for improvements in fruit and vegetable consumption (0.4 servings; *p* < 0.001) [105]. A study using visual estimation in two elementary schools in Minnesota conducted by Burgess-Champoux and colleagues focused on whole grains. This intervention included five lessons, cafeteria staff culinary training (e.g., quality control, staff taste tests, etc.) and a parent component (e.g., supermarket/bakery tours, newsletters). They found an average of a one serving increase in whole grain consumption (*p* < 0.0001) [106]. In a study conducted by Head, marginally significant (*p* = 0.05) increases in overall consumption were observed using weighed plate waste in four North Carolina elementary schools exposed to nutrition education with a focus on basic nutrition and dietary patterns [107]. Additionally, higher rates of vegetable consumption were observed using digital imagery among 6th grade students exposed to a curriculum that focused on sustainable food systems in a study conducted by Prescott et al. [108]. Increases in selection were also observed in the intervention schools in this study, however they were not statistically significant. Lastly, greater consumption was also observed in four previously described multi-component studies that combined nutrition education with cafeteria components including increases in fruit and vegetable choices [47,52] and taste tests [73,74].

Inconsistent results were found in a multi-component study conducted by Blakeway et al. that included both nutrition education and taste tests; improvements were observed in some, but not all, grades [75]. Additionally, mixed findings were observed by Liquori and associates with a nutrition education program in two New York City elementary schools; while lessons that focused on food and the environment had no impact on consumption (using visual estimation), nutrition education that included a cooking component improved consumption of vegetables and whole grains, but only among younger students [50]. Jones et al. also evaluated a curriculum that involved both nutrition and agriculture, as well as farm to school activities (e.g., school gardens, field trips, cooking demonstrations) in 18 elementary and middle schools in South Carolina and found a small decrease in fruit consumption (−0.07 servings; *p* < 0.05) and no significant impact on vegetable consumption using digital imagery [109]. Another study conducted by Ishdorj et al. examined lunch consumption based on 24-hour recalls from a nationally representative sample of public schools and found that nutrition education was not associated with fruit or vegetable consumption [110]. No impact was observed in four other studies conducted in elementary schools with nutrition education that all specifically focused on fruit and vegetable consumption, with intake measured using visual estimation [85,111,112] or digital imagery [113]. Lastly, no associations were found in three other previously described multi-component studies that incorporated both nutrition education and taste tests [76,77,78]. 

#### 3.2.3. School Lunch Duration

Of the four peer-reviewed publications that examined school lunch duration, three found a positive association between duration and school meal consumption and one found no association (Table 4). Three studies had a low risk of bias and one had a high risk of bias; of the studies with a low risk of bias, two found a positive association with school meal consumption.

In a study conducted by Bergman et al. using weighed plate waste in two elementary schools in Washington, students with a 30 min lunch period consumed on average 72.8% of their meal, while students who had a 20 min lunch period consumed on average only 56.6% of their meal (*p* < 0.0001) [114]. In a similar study conducted by Cohen and colleagues using weighed plate waste in six elementary and middle schools in Massachusetts, students who had less than 20 min of seated time for lunch consumed on average between 10–13% less of their meal compared with students who had more than 25 min to eat lunch (corresponding with a 30 min lunch period; *p* < 0.0001) [19]. This study also found that students were more likely to select a fruit when they had more time to eat. Using digital imagery to assess consumption, Gross et al. examined 10 elementary schools in New York City and found that lunch periods that were at least 30 min long were associated with an increased odds of consuming fruits (OR = 2.0; *p* = 0.02) and whole grains (OR = 2.1; *p* < 0.05) [115]. Lastly, in another study conducted in New York City among elementary school students, Ang et al. found that lunch period duration was not associated with consumption [51]. However, less than 15% of the students had schedules with lunch periods greater than 20 min, which may have limited the ability to detect significant differences. 

#### 3.2.4. Recess before Lunch 

Of the 10 peer-reviewed publications that examined recess before lunch (Table 4), seven found a positive association with school meal consumption, one found mixed results with different meal components, and two found no association. Of the studies examining the timing of recess that were considered to have a low risk of bias, the majority (3 out of 4) found positive associations with school meal consumption for differing meal components.

Seven studies examining recess before lunch used weighed plate waste methodology. Bergman and colleagues examined two elementary schools in Washington and found that students who had recess before lunch consumed on average 72.8% of their meal compared with students who had recess after lunch and consumed on average 59.9% of their meal (*p* < 0.0001) [116]. In a study conducted by Chapman et al. among students attending eight elementary schools in New Orleans, recess before lunch was associated with a 5% increase in fruit consumption, but no differences were observed for other meal components [117]. This study also found that students with lunch periods early or late in the day consumed less than students with traditional lunch hours. Getlinger and associates also found overall reduction in food waste from 34.9% to 24.3% in one elementary school in Illinois [118] Strohbehn et al. used a combination of weighed plate waste and digital imagery in three elementary schools, similarly finding reduced waste for fruits, as well as grains and meat/meat alternatives, but found increases in waste for vegetables [119]. Two additional studies, conducted by Hunsberger et al. in one elementary school in Oregon and McLoughlin et al. in two elementary schools in Illinois, found recess before lunch was associated with greater milk consumption, but found no association with other meal components [21,120]. Lastly, Tanaka et al. examined one elementary school in Hawaii and found no association with school meal consumption using weighed plate waste [121]. Among studies using other consumption measures, research conducted by Ang et al. among elementary students in New York City examined consumption with visual estimation and found recess before lunch was associated with small increases in the amount of fruits (0.08 cups; *p* < 0.001) and vegetables (0.007 cups; *p* = 0.04) eaten [51]. Another study conducted by Price et al. in seven elementary schools in Utah using visual estimation to assess diet also found that recess before lunch was associated with greater fruit and vegetable consumption (0.16 servings; *p* < 0.01) [122]. However, a study conducted by Fenton and associates using diary assisted 24-hour recalls among students attending 31 elementary schools in California found no association between the timing of recess and fruit and vegetable consumption at lunch [123].

### 3.3. Policies 

#### 3.3.1. Federal Policies: The Healthy Hunger Free Kids Act (HHFKA) 

Out of the six studies that examined the impact of the HHFKA, most (4 of 6) found increases or no impact on consumption, with two studies finding a decrease in intake for certain meal components (Table 5). Among the studies with a low risk of bias, two out of three found that this federal policy was associated with increases in school meal consumption. 

Cohen and colleagues measured consumption using weighed plate waste in four elementary and K-8 schools in Massachusetts and found a 15.6% increase in entrée consumption (*p* < 0.0001) and a 16.2% increase in vegetable intake (*p* < 0.0001) [24]. Additionally, significantly more students selected fruit, resulting in more students consuming this meal component. Consistent results were found by Schwartz et al. in 12 middle schools using the same measure of consumption; entrée consumption increased from 71% to 84% (*p* < 0.05) and vegetable consumption increased by roughly 20% (*p* < 0.05) [25]. Greater fruit selection was also observed in this study. Ishdorj and colleagues examined consumption using aggregate plate waste in three Texas elementary schools, and found no impact on consumption [98]. Similarly, Bontranger Yoder et al. found no association with school meal intake using digital imagery in 11 elementary schools in Wisconsin [78]. Cullen et al. found no association with fruit, vegetable, or entrée consumption, and a decrease in milk consumption using visual estimation after implementation of the HHFKA, although they observed significant increases in the selection of fruits, vegetables, legumes, and protein foods [26]. Contrasting with the results of the other studies, Amin et al. found a small decrease in fruit and vegetable consumption by roughly 0.05 cups (*p* < 0.01) in two elementary schools (using both digital imagery and weighed plate waste) after the HHFKA went into effect, although increases in fruit selection were observed [124]. 

#### 3.3.2. Local and State Policies: Access to Competitive Foods 

Six studies examined the impact of limiting student access to competitive foods (either via state or local policies or based on student purchases) and nearly all (5 of 6) found that consumption of school meals was inversely associated with access to competitive foods. Only one study had a low risk of bias and found that students consumed more of their healthier school meals when access to competitive foods was limited.

Cullen and colleagues examined Texas elementary schools without access to competitive foods and a middle school where students could purchase snacks/beverages and observed that the elementary students without access to competitive foods consumed significantly more fruits and vegetables than the middle school students based on self-reported measures of intake [125]. Additionally, they found greater selection of fruit, vegetables, and milk when students did not have access to competitive foods or beverages. Similarly, Cullen and colleagues examined the transition from elementary school to middle school and found that students consumed significantly fewer fruits and vegetables and less milk when they entered middle school and had new access to competitive foods [126]. Marlette et al. used weighed plate waste in three Kentucky middle schools and found similar decreases in school meal consumption when students purchased competitive foods [127]. Using a nationally representative sample with 24-hour recall data, Ishdorj et al. found that students attending schools with policies that placed restrictions on the sale of competitive foods and desserts reported greater vegetable consumption. They also found that when policies were in place limiting access to higher fat milk (whole or 2%), students reported increases in both fruit and vegetable consumption. In contrast, students reported reduced fruit intake when there were policies limiting French fries [110]. When Cullen and associates used lunch food records to examine a Texas state policy that strengthened the standards for competitive foods, they found that students in three middle schools reported greater vegetable and milk consumption [128]. Of note, when a local policy in Texas only limited access to vending machines in the cafeteria (but vending machines with competitive foods were available in other parts of the school), Cullen et al. observed that students in three middle schools compensated by increasing their competitive food purchases from the other vending machines. Students also increased their milk consumption but decreased their vegetable consumption [129].

#### 3.3.3. Other Local Policies

Limiting flavored milk is a policy that has been addressed in some locations. Four studies have examined the impact of local policies limiting access to chocolate milk, and among the two studies with a low risk of bias, one found increases in waste and one found no impact. Among the three studies examining other local policies, two studies with a high risk of bias found an increase in consumption and one study with a low risk of bias had mixed findings. 

Cohen and colleagues examined weighed plate waste in four elementary/K-8 schools in Massachusetts where access to chocolate milk was limited and found a 10% decline in unflavored milk consumption within the first year of implementation (as well as reduced selection of milk) [24]. Greater unflavored milk waste was also observed by Hanks et al. using visual estimation in 25 elementary schools after chocolate milk was removed [130]. Among the studies with longer exposure to the policy (i.e., two years), Cohen et al. found that students adjusted to the policy and there was no adverse impact on average milk intakes (nor selection) in four Boston middle schools using weighed plate waste [56]. While Schwartz et al. found that individual-level consumption of milk was lower using weighed plate waste in two K-8 schools after two years of exposure to a policy that removed chocolate milk, importantly this study found that school-wide per-student consumption significantly increased from 2.1oz to 2.5oz due to the large increases in selection (from 51.5% to 72%) [131]. Interestingly, this study also found that milk consumption was adversely impacted by offering juice on the lunch menu. 

Another practice included in local policies is serving breakfast in the classroom. Farris et al. examined breakfast in the classroom within seven elementary schools in Virginia and found that the policy was associated with decreases in food waste from 43% to 38.5% of overall meals discarded [132]. Another study mentioned earlier assessed the impact of a wellness policy that included provisions for more fruit and vegetable choices, as well as an increase in contact hours for health and physical education. This was associated with increases in fruit and vegetable consumption [47]. Lastly, a study conducted by Canterberry and associates in seven elementary schools in New Orleans assessed a local policy that required an increase in fresh and less-processed ingredients, as well as a restriction on deep-fried foods. There were three food service providers and the findings were mixed: some lower intakes were observed in the schools with one of the food service providers, but there were no associations with consumption in the schools with the two other food service providers [133]. 

**Table 1:** Characteristics and outcomes of studies examining initiatives and interventions targeting meal-level factors included in the systematic review.

Author, Year	Location; Participant Characteristics	Study Design ^1^	Year(s)	Exposure(s)	Outcome Measure(s)	Results	**Risk of Bias ^2^**
Choices							
Adams et al. 2005 [55]	San Diego, CA; 2 school districts (2 elementary schools per district [288 students total])	QE: Post-only (with comparison group)	2003	Choices: Salad bar and pre-portioned options with varying number of options were provided	Weighed plate waste	Selection: There were no significant associations. Consumption: The presence of a salad bar alone was not associated with F/V selection or consumption. However, a greater number of F/V options was associated with a trend in increased F/V consumption.	High
Ang et al. 2019 [51]	New York City, NY; 14 elementary schools [877 trays collected from students in grade 2–3)	QE: Post-only (with comparison grou**p**)	2015–16	(1) Choice Architecture: Vegetables were pre-plated (vs. optional), and were placed first in line (2) Pre-sliced Fruit (3) Recess before lunch (4) Choices: multiple (2+) fruit and vegetable options were provided (5) Lunch Duration	Visual estimation	Selection: Not measured Consumption: Pre-plating vegetables (vs. optional for the student to select a vegetable) was associated with a small increase in consumption (0.02 cups; *p* < 0.001). Positioning vegetables first on the serving line was not associated with vegetable consumption. Among students who selected fruit, pre-sliced fruit was associated with greater consumption (0.23 cups more; *p* = 0.02) than whole fruit.. Recess before lunch was associated with a small increase in fruit consumption (0.08 cups; *p* < 0.001) and vegetable consumption (0.007 cups; *p* = 0.04).Multiple fruit options and attractive serving bowls were not associated with fruit consumption. Lunch duration was not associated with consumption (although less than 15% of measurements had lunch durations of ≥20 min).	Low
Bean et al. 2018 [53]	VA; 2 elementary schools (725 trays collected from students in grades 1–5)	QE: Pre/post (no comparison group)	2015–16	Choices: Salad bars were added to the serving line with additional fruit and vegetable options	Digital imagery	Selection: Salad bars were associated with an increase in the number of F/V selected (1.81 vs. 2.58 F/V; *p* < 0.001). Consumption: Salad bars were associated with a decrease in F/V consumption by 0.65 cups (*p* < 0.001).	High
Cullen et al. 2015A [44]	Houston, TX; 8 elementary schools and 4 middle schools (1576 trays from students in grades K-8)	QE: Post-only (with comparison group)	Fall 2011	Choices: Students in intervention schools were allowed to select one fruit serving and two vegetables servings (three total). Students in comparison schools were limited to no more than two servings of fruits and/or vegetables	Visual estimation	Selection: In the intervention elementary schools, there was significantly greater selection of fruits and starchy vegetables and decreased selection of juice compared with control schools. In the intervention middle schools, there was significantly greater selection of fruit, total vegetables, and starchy vegetables compared with control schools.Consumption: In the intervention elementary schools, students consumed more vegetables compared than the comparison group (0.14 cups vs. 0.10 cups; *p* < 0.01). There was no impact on whole fruit consumption. In the intervention middle schools, students increased their consumption of both vegetables (0.17 cups vs. 0.10; *p*<0.01) and whole fruits (0.19 vs. 0.09; *p* < 0.001).	High
Greene et al. 2017 [49]	NY; 7 middle schools (8502 trays from students in grades 5–8)	RCT	2014	(1) Choice Architecture: Fruits were placed first on the lunch line and were in attractive bowls with descriptive names. Promotional materials (e.g., fruit facts) were posted in the cafeteria (2) Choices: Multiple (2) fruit and vegetable options were provided (3) Pre-sliced Fruit	Visual estimation	Selection: The intervention was associated with a 36% increase in fruit selection (*p* < 0.001), but no significant changes in vegetable or milk selection. Consumption: The intervention was associated with a 23% increase in fruit consumption (*p* < 0.001). There was no association with vegetable or milk consumption.	Low
Hakim et al. 2013 [46]	Midwest Region; 1 K-8 school (2148 trays from students in grades K-8)	QE: Pre/Post (no comparison group)	2011–2012	Choices: Students were provided with a choice of three F/V options	Weighed plate waste and visual estimation	Selection: Not measured Consumption: The intervention was associated with a 15% increase in fruit consumption and a 16% increase in vegetable consumption.	High
Johnson et al. 2017 [54]	New Orleans, LA; 21 middle and high schools (718 students in grades 7–12)	QE: Post-only (with comparison group)	N.S.	Choices: Salad bars were added	24-hour recall (interviewer assisted ASA-24 Kids)	Selection: Not measured Consumption: There were no significant differences in vegetable consumption. Among students who reported consuming any fruit, students in schools with salad bars reported lower levels of fruit consumption at lunch compared to students in schools without salad bars.	High
Just et al. 2012 [45]	N.S.; 22 elementary schools (48,533 trays)	Cross-sectional	N.S.	Choices: The number of fruit and vegetables options available to students was assessed	Visual estimation	Selection: Not measured Consumption: Each additional fruit or vegetable option offered increased the fraction of students who ate at least one serving of fruits and vegetables by 12%.	High
Liquori et al. 1998 [50]	New York City, NY; 2 elementary schools (590 students in grades K-6)	QE: Pre/post (with comparison group)	1995–96	(1) Choices: The number of vegetable and whole grain options available was increased (2) Nutrition Education: 10 food and environment lessons and/or 10 cooking lessons were provided (3) Other: Students took field trips to a local community garden. There was parent outreach (newsletter, recipes, workshops)	Visual estimation	Selection: Not measured Consumption: The intervention arm with cooking lessons was associated with increased consumption of vegetables and whole grains among younger students (*p* < 0.01). No association was observed among older children exposed to the cooking intervention. The nutrition education (food environment) intervention was not associated with consumption.	High
Perry et al. 2004 [48]	St. Paul, MN; 26 elementary schools (1,668 students in grades 1 and 3)	RCT	2000–2002	(1) Choice Architecture: Verbal prompts by food service staff encouraging consumption; improved attractiveness of F/V (e.g., placing them in small cups; arranging by color); posters and characters (life size fruit and vegetables); and rewards for eating F/V (classroom receives frozen fruit yogurt if enough students ate 3 servings at lunch) (2) Choices: The quantity of F/Vs was increased (3) Taste Tests	Visual estimation	Selection: Not measured Consumption: The intervention was associated with significant increases in total servings of F/V (excluding potatoes and juice).	Low
Taylor et al. 2018 [52]	CA; 2 elementary schools (112 students in grade 4)	QE: Pre/post (with comparison group)	2012–13	(1) Choices: Salad bars were added to increase F/V choices (2) Nutrition Education: A garden enhanced nutrition curriculum and cooking demonstrations were provided (3) Other: Parent newsletter, home activities	Digital imagery	Selection: No significant associations Consumption: The intervention was associated with a significant increase in vegetable consumption. There was no association with fruit consumption.	High
Young et al. 2013 [47]	N.S.; 1 middle school (3810 trays from students in grades 6-8)	Cross-Sectional	2011–12	Policy: A new wellness policy required schools to implement the practices below: (1) Choices: Different fruits and vegetables were served each day of the week (2) Nutrition Education: There was an increase in student contact hours for health and physical education	Visual estimation	Selection: Not measured Consumption: After exposure to the wellness policy for over a semester, students consumed significantly more fruits and cooked vegetables.	High
**Food Preparation: Palatability and Cultural Appropriateness**							
Bates et al. 2015 [64]	UT; 2 schools (1 middle and 1 high school [2760 school breakfasts from students in grades 7–12]	QE: Pre/post (no comparison group)	Not Stated	Palatability: Smoothies made with whole fruit, yogurt, and milk or fruit juice were offered to students	Visual estimation	Selection: Not measured Consumption: Offering breakfast smoothies was associated with a 0.45 serving (*p* < 0.01) increase in fruit consumption.	High
Cohen et al. 2012 [56]	Boston, MA; 4 middle schools (3049 students in grades 3–8)	QE: Post-only (with comparison group)	2009	(1) Palatability: A professional chef trained cafeteria staff to prepare healthier school lunches (2) Policy: Chocolate milk was removed	Weighed plate waste	Selection: The intervention was associated with a 51% increase in whole grain selection (*p* = 0.02). Consumption: Students in the intervention schools consumed 0.36 more servings of vegetables per day (*p* = 0.01) compared with students in control schools. There was no impact on milk, fruit, or whole grain consumption.	Low
Cohen et al. 2015 [57]	MA; 14 elementary and middle schools (2638 students in grades 3–8)	RCT	2011–12	(1) Choice architecture: Vegetables were offered at the beginning of the lunch line; fruits were placed in attractive containers; fruits were placed next to the cash register; signage added that promoted F/V; white milk was placed in front of chocolate milk (2) Palatability: A professional chef trained cafeteria staff to prepare healthier school lunches	Weighed plate waste	Selection: Both the choice architecture and chef (i.e., palatability) intervention were associated with increased fruit and vegetable selection. There was no impact on white milk selection. Consumption: Only the chef intervention was associated with increased consumption of fruits and vegetables. There was no impact on white milk consumption.	Low
Cohen et al. 2019 [58]	MA; 8 elementary and middle schools (1309 students in grades 3–8)	QE: Pre/post (with comparison group)	2012-13	Palatability: A professional chef trained cafeteria staff to prepare healthier school lunches	Weighed plate waste	Selection: No significant associations Consumption: The intervention was associated with a significant increase in consumption of vegetables (62.2% vs. 38.2%; *p* = 0.005) and fruits (75.2 vs. 59.2%; 0 = 0.04) compared with control schools. There were no significant differences in entrée consumption.	Low
D’Adamo et al. 2021 [61]	Baltimore, MD; 1 high school (4570 trays from students in grades 9–12)	QE: Pre/post (no comparison group)	N.S.	Palatability: Spices and herbs were added to vegetable recipes (based in prior pilot taste tests with some students)	Weighed plate waste	Selection: Not measured Consumption: The intervention was associated with an 18.2% increase in vegetable consumption (*p* < 0.001).	High
Fritts et al. 2019 [62]	PA; 1 middle/high school (~600–700 students ages 11–18)	QE: Pre/post (no comparison group)	2017	(1) Palatability: Spices and herbs were added to vegetable recipes (2) Other: Repeated (i.e., 5) exposures to vegetables	Weighed plate waste	Selection: Not measured Consumption: The intervention was inversely associated with consumption for some of the vegetables offered. There was no association between repeated exposures to vegetables with added spices/herbs and vegetable consumption.	High
Hamdi et al. 2020 [63]	IL; 3 elementary schools (1255 trays from students in grades K-8)	QE: Pre/post (no comparison group)	2018–19	(1) Choice Architecture: Cafeteria decorations and creative names were introduced (2) Taste Tests (3) Palatability: A flavor station with spices and seasonings on a table in the cafeteria was added	Weighed plate waste	Selection: Selection was measured in only one of the participating schools. The odds of selecting a vegetable (i.e., broccoli) increased when students were exposed to taste tests. The odds of selecting fruit increased when all intervention components were implemented simultaneously. Consumption: The intervention components yielded inconsistent, but generally positive consumption results across the schools, particularly for fruits.	Low
Just et al. 2014 [60]	NY; 1 High School (3330 trays)	QE: Pre/post (no comparison group)	2012	(1) Palatability: Chef enhanced recipes for pizza were introduced (2) Taste Tests	Visual estimation	Selection: Student selection of chef-enhanced entrées increased by 5.7% percentage points (91.3% to 97%; *p* = 0.01). Student selection of salad, which was served with the pizza, increased by 21% percentage points (*p* < 0.001). Consumption: The chef enhanced pizza was not associated with differences in main dish consumption. However, as more students selected salad with the pizza, vegetable consumption increased by 16.5% (*p* = 0.005).	High
Zellner et al. 2017 [59]	Philadelphia, PA; 2 elementary schools (students in grades 3–4)	QE: Pre/post (with comparison group)	N.S.	(1) Palatability: Chef prepared meals were introduced (2) Other: Meals were served family-style and an adult (i.e., teacher) ate with students as a role model	Visual estimation	Selection: Not measured Consumption: The intervention was associated with an increase in sweet potato fry consumption at the beginning of the school year and an increase in cauliflower consumption at the end of the school year.	Very High
**Food Preparation: Pre-Slicing Fruit**							
Ang et al. 2019 [51]	New York City, NY; 14 elementary schools [877 trays collected from students in grade 2–3)	QE: Post-only (with comparison grou**p**)	2015–16	(1) Choice Architecture: Vegetables were pre-plated (vs. optional), and were placed first in line (2) Pre-sliced Fruit (3) Recess before lunch (4) Choices: multiple (2+) fruit and vegetable options were provided (5) Lunch Duration	Visual estimation	Selection: Not measuredConsumption: Pre-plating vegetables (vs. optional for the student to select a vegetable) was associated with a small increase in consumption (0.02 cups; *p* < 0.001). Positioning vegetables first on the serving line was not associated with vegetable consumption Among students who selected fruit, pre-sliced fruit was associated with greater consumption (0.23 cups more *p* = 0.02) than whole fruit. Recess before lunch was associated with a small increase in fruit consumption (0.08 cups; *p* < 0.001) and vegetable consumption (0.007 cups; *p* = 0.04). Multiple fruit options and attractive serving bowls were not associated with fruit consumption. Lunch duration was not associated with consumption (although less than 15% of measurements had lunch durations of ≥20 min).	Low
Greene et al. 2017 [49]	NY; 7 middle schools (8502 trays from students in grades 5-8)	RCT	2014	(1) Choice Architecture: Fruits were placed first on the lunch line and were in attractive bowls with descriptive names. Promotional materials (e.g., fruit facts) were posted in the cafeteria (2) Choices: Multiple (2) fruit and vegetable options were provided (3) Pre-sliced Fruit	Visual estimation	Selection: The intervention was associated with a 36% increase in fruit selection (*p* < 0.001), but no significant changes in vegetable or milk selection. Consumption: The intervention was associated with a 23% increase in fruit consumption (*p* < 0.001). There was no association with vegetable or milk consumption.	Low
McCool et al. 2005 [66]	N.S.; 1 elementary and 1 middle school	QE: Pre/post (no comparison group)	N.S.	Pre-sliced Fruit: Sliced apples and/or whole apples	Aggregate plate waste	Selection: Not measured Consumption: Elementary school students consumed more fruit when apples were pre-sliced than when they were served whole. Middle school students consumed more fruit when they had a choice between pre-sliced or whole apples.	Very High
Quinn et al. 2018 [70]	King County, WA; 11 middle and high schools (2309 trays)	QE: Pre/post (with comparison group)	2013–14	(1) Pre-sliced Fruit: (2) Choice Architecture: Changes included attractive containers, creative names, signage, food placement (e.g., multiple locations and/or at eye-level), and verbal prompts by cafeteria staff	Visual estimation	Selection: A greater proportion of students selected fruit in the intervention schools compared with control schools. There was no significant change in vegetable selection. Consumption: Pre-sliced fruit and choice architecture were not associated with significant differences in the quantities of fruits, vegetables, or milk consumed.	High
Smathers et al. 2020 [65]	N.S.; 2 elementary schools (students in grades PreK-5)	QE: Pre/post (no comparison group)	N.S.	Pre-sliced Fruit: Sliced apples were compared with whole apples	Aggregate weighed plate waste	Selection: No significant associations Consumption: Students consumed 2.48 times more fruit by weight when eating pre-sliced versus whole apples (*p* < 0.001).	Very High
Swanson et al. 2009 [68]	KY; 1 elementary school (979 students in grades K-4)	QE: Pre/post (no comparison group)	2007	Pre-sliced Fruit: Sliced apples and oranges were compared with whole apples and oranges	Digital imagery	Selection: Pre-slicing oranges was associated with increased selection. No significant differences were observed with pre-sliced apples. Consumption: A greater proportion of students consumed at least half of a fruit serving when pre-sliced (vs. whole) oranges were served. There were differences observed by grade level (i.e., higher consumption rates among younger students with pre-sliced oranges). The intervention was not associated with consumption for apples.	High
Thompson et al. 2017 [69]	Hennepin County, MN; 2 elementary schools (373 students in grades K-4)	QE: Pre/post (no comparison group)	2013	(1) Choice Architecture: Changes included enhanced displays for F/V; attractive labels for F/V; and placement of F/V at the beginning of the lunch line and at the cash register (2) Pre-sliced Fruit: Sliced apples were compared with whole apples	Weighed plate waste	Selection: The intervention was associated with a significant increase in the percentage of students selecting a fruit serving (95.1% vs. 98.1%; *p* = 0.02). There was no significant change in vegetable selection. Consumption: The intervention was not associated with fruit or vegetable consumption.	Low
Wansink et al. 2013 [67]	Wayne County, NY; 6 middle schools (334 trays)	RCT	2011	Pre-sliced Fruit: Sliced apples were compared with whole apples	Visual estimation	Selection: There was a significant increase in apple selection in intervention schools when pre-sliced apples were offered (5% difference in sales between intervention and comparison schools; *p* < 0.001). Consumption: Pre-slicing apples was associated with an increase in the percent of students who selected an apple and ate more than half (75% increase; *p* = 0.02).	Low
**Taste Tests**							
Alaimo et al. 2015 [73]	Grand Rapids, MI; 6 elementary schools (4 intervention and 2 control 815 students in grades 3–5)	QE: Pre/post (with comparison group)	2009–10 to 2010–11	(1) Taste Tests: Provided in the cafeteria and classrooms (2) Nutrition Education: Nutrition education classes and posters (3) Other: Healthy eating coaching by teachers, parent education	Digital imagery	Selection: Not measured Consumption: The multi-component intervention was associated with significant increases in fruit consumption. No differences in consumption of vegetables, milk, grains, or protein were observed.	Low
Blakeway et al. 1978 [75]	Little Rock, AR; 16 elementary schools (5000 students in grades 1-3)	RCT	N.S.	(1) Taste Tests: Provided in the classroom with nutrition education (2) Nutrition Education: A nutrition coordinator implemented 10 classroom lessons focused on recognizing and identifying foods in different forms.	Aggregate plate waste	Selection: Not measured Consumption: Students in intervention schools consumed greater amounts of whole wheat rolls (grades 2 and 3 only) and cottage cheese (grades 1 and 2 only) compared to the comparison group. Sweet potato custard consumption increased in both the intervention and control group. No other significant differences were observed.	High
Bontranger Yoder et al. 2014 [77]	WI; 9 elementary schools (1117 students in grades 3–5)	QE: Pre/post (no comparison group)	2010–11	(1) Nutrition Education: A farm to school gardening curriculum was available in some of the participating schools (2) Taste tests: Available in some of the participating schools (3) Other: Farm to school activities were introduced (e.g., a school garden, field trips to farms, and local items on the menu) in some of the participating schools	Digital imagery	Selection: Not measured Consumption: The intervention was not associated with differences in F/V consumption, although the farm to school components were inconsistently implemented across the participating schools.	Low
Bontranger Yoder et al. 2015 [78]	WI; 11 elementary schools (7117 trays from students in grades 3–5)	Cross-sectional and Pre/post (no comparison group)* *For Policy only	2010 to 2013	(1) Nutrition Education: A farm to school gardening curriculum was available in some of the participating schools (2) Taste tests: Available in some of the participating schools (3) Policy: HHFKA (4) Other: Farm to school activities were introduced (e.g., a school garden, field trips to farms, and local items on the menu) in some of the participating schools	Digital imagery	Selection: Not measured Consumption: The intervention components (i.e., nutrition education, taste tests, and other activities) were not associated with differences in F/V consumption. There was no change in consumption before or after implementation of the HHFKA.	High
Georgiou (1998 Gov’t Report) [74]	OR; 1 elementary school (40 students in grade 3)	QE: Pre/post (with comparison group)	1997	(1) Nutrition Education: Lessons included the Food Guide Pyramid, healthy meal planning, and how foods grow (2) Taste Tests: Provided in the classroom with nutrition education	Weighed plate waste	Selection: No significant associations Consumption: The intervention was associated with an increase in consumption of calories from fruits (28 kcal; *p* < 0.01). No significant differences in vegetable and grain consumption were observed.	High
Hamdi et al. 2020 [63]	IL; 3 elementary schools (1255 trays from students in grades K-8)	QE: Pre/post (no comparison group)	2018–19	(1) Choice Architecture: Cafeteria decorations and creative names were introduced (2) Taste Tests (3) Palatability: A flavor station with spices and seasonings on a table in the cafeteria was added.	Weighed plate waste	Selection: Selection was measured in only one of the participating schools. The odds of selecting a vegetable (i.e., broccoli) increased when students were exposed to taste tests. The odds of selecting fruit increased when all intervention components were implemented simultaneously. Consumption: The intervention components yielded inconsistent, but generally positive consumption results across the schools, particularly for fruits.	Low
Just et al. 2014 [60]	NY; 1 High School (3330 trays)	QE: Pre/post (no comparison group)	2012	(1) Palatability: Chef enhanced recipes for pizza were introduced (2) Taste Tests	Visual estimation	Selection: Student selection of chef-enhanced entrées increased by 5.7% percentage points (91.3% to 97%; *p* = 0.01). Student selection of salad, which was served with the pizza, increased by 21% percentage points (*p* < 0.001). Consumption: The chef enhanced pizza was not associated with differences in main dish consumption. However, as more students selected salad with the pizza, vegetable consumption increased by 16.5% (*p* = 0.005).	High
Mazzeo et al. 2017 [71]	Mid-Atlantic region; 2 elementary schools (2087 trays from students in grades 1–3)	QE: Pre/post (with comparison group)	2014–15	(1) Choice Architecture: Students were rewarded for eating F/V (i.e., sticker and praise) (2) Taste Tests	Visual estimation	Selection: Not measured Consumption: The intervention was associated with reduced F/V waste.	High
Morrill et al. 2016 [72]	UT; 6 elementary schools (2292 students in grades	QE: Pre/post (with comparison group)	2011	(1) Choice Architecture: Students were rewarded for eating F/V (i.e., toys or praise from teachers). Adults provided role modeling (2) Taste Tests (3) Other: Videos and letters	Visual estimation	Selection: Not measured Consumption: The intervention was associated with increased F/V consumption. At 6 months follow-up (after the intervention ended), only the intervention arm with prizes (plus nutrition education and taste tests) was associated with sustained increased consumption.	Low
Perry et al. 2004 [48]	St. Paul, MN; 26 elementary schools (1668 students in grades 1 and 3)	RCT	2000–2002	(1) Choice Architecture: Verbal prompts by food service staff encouraging consumption; improved attractiveness of F/V (e.g., placing them in small cups; arranging by color); posters and characters (life size fruit and vegetables); and rewards for eating F/V (classroom receives frozen fruit yogurt if enough students ate 3 servings at lunch) (2) Choices: The quantity of F/Vs was increased (3) Taste Tests	Visual estimation	Selection: Not measured Consumption: The intervention was associated with significant increases in total servings of F/V (excluding potatoes and juice).	Low
Reynolds et al. 2000 [76]	AL; 28 elementary schools (425 students in grade 4)	QE: Pre/post (with comparison group)	1994 to 1996	(1) Nutrition Education: A 14 lesson curriculum related to F/V was provided and posters were added to the cafeteria (2) Taste Tests (3) Other: Parent education was provided, including recipes, activities, and information about F/V	Visual estimation	Selection: Not measured Consumption: The intervention was not associated with significant differences in fruits or vegetables consumed.	Low

F/V: fruits and vegetables; QE: Quasi-Experimental; RCT: Randomized controlled trial. ^1^ Study designs were defined as: (1) Cross-sectional- Observational study with the exposure and outcome measured simultaneously (no comparison group); (2) Quasi-Experimental (QE): Post Only—Intervention study with a comparison group and data collected post-implementation (no baseline measurements); (3) Quasi-Experimental (QE): Pre/Post— Intervention studies with pre-implementation (i.e., baseline) and post-implementation measurements, with or without a comparison group (non-random allocation of intervention/comparison groups); (4) Randomized Controlled Trial (RCT)—Intervention study with random allocation to intervention or control status and both pre-implementation (baseline) and post-implementation measurements. ^2^ Risk of Bias was based on adapted Newcastle–Ottawa Scales (NOS) for cross-sectional and cohort studies (Supplementary Materials Table S1).

**Table 2:** Characteristics and outcomes of studies examining initiatives and interventions targeting cafeteria environment-level choice architecture techniques included in the systematic review.

Author, Year	Location; Participant Characteristics	Study Design ^1^	Year(s)	Exposure(s)	Outcome Measure(s)	Results	**Risk of Bias ^2^**
Choice Architecture: Rewards							
Blom-Hoffman et al. 2004 [85]	Northeast Region; 1 elementary school (students in grades K-1)	RCT	N.S.	(1) Choice Architecture: Students received verbal praise and rewards (i.e., stickers) for consuming F/V (2) Nutrition Education: 10 lessons with 5-A-Day information was implemented by the classroom teacher and a school psychology graduate student (3) Other: Parent component (newsletter and cookbook) included	Visual estimation	Selection: Not measured Consumption: The intervention was not associated with differences in vegetable consumption.	High
Hendy et al. 2005 [79]	PA; 1 elementary school (188 students in grades 1, 2, and 4)	QE: Pre/post (no comparison group)	N.S.	Choice architecture: Students received a reward for eating F/V (small prize for eating ≥1/8 cup)	Visual estimation	Selection: Not measured Consumption: The reward intervention was associated with an increase in consumption for both fruits and vegetables.	High
Hoffman et al. 2010 [83]	New England region; 4 elementary schools (297 students in grades 1 and 2)	RCT	2006 to 2007	(1) Choice architecture: Students received verbal encouragement for eating F/V; a reward for tasting F/V (i.e., a sticker); and promotional posters with cartoon characters (2) Other: There were school wide promotions (announcements about the F/V of the day); a classroom component (computer game with 5-a-Day messaging); and a family component (i.e., cookbook, interactive children’s books)	Weighed plate waste	Selection: Not measured Consumption: The intervention was associated with an increase in fruit consumption in both year 1 (29 g difference; *p* < 0.0001) and year 2 (21 g difference; *p* < 0.005), and an increase in vegetable consumption in year 1 (6 g difference; *p* < 0.01). No significant difference in vegetable consumption was observed in year 2.	Low
Hoffman et al. 2011 [84]	New England region; 4 elementary schools (297 students in grades 1 and 2)	RCT	2005 to 2009	(1) Choice architecture: Students received verbal encouragement for eating F/V; a reward for tasting F/V (i.e., a sticker); and promotional posters with cartoon characters (2) Other: There were school wide promotions (announcements about the F/V of the day); a classroom component (computer game with 5-a-Day messaging); and a family component (i.e., cookbook, interactive children’s books)	Weighed plate waste	Selection: Not measured Consumption: The intervention was associated with an increase in F/V at lunch during the study period, but at one year follow-up (after the intervention concluded), there was no longer a difference in F/V consumption.	Low
Hudgens et al. 2017 [86]	Cincinnati, OH; 1 elementary school (207 trays from students in grades K-6)	QE: Pre/post (no comparison group)	2014–15	Choice Architecture: Students received emoticons and rewards (small prizes) for selecting a lunch with a fruit, vegetable, unflavored milk, and whole grain	Visual estimation	Selection: The intervention was associated with a significant increase in the selection of plain fat-free milk and vegetables, and a decrease in the selection of flavored milk. Consumption: The intervention was not associated with differences in consumption for any meal component.	High
Jones et al. 2014 [80]	Logan, UT: 1 elementary school (K-5)	QE: Pre/post (no comparison group)	2013	Choice Architecture: Students received rewards (i.e., virtual currency or teacher continuing to read a story) for eating FV. Students also received teacher encouragement to eat more FV when average consumption levels were lower	Aggregate plate waste	Selection: Not measured Consumption: On days when fruits were targeted, fruit consumption was 39% higher (*p* < 0.01), but there was no change in vegetable consumption. On days when vegetables were targeted, vegetable consumption was 33% higher (*p* < 0.05), but there was no change in fruit consumption.	Very High
Machado et al. 2020 [82]	OR; 1 elementary school (797 trays from students in grades K-5)	QE: Pre/post (no comparison group)	2016–17	Choice Architecture: Elements included adult role modeling, verbal prompts, and rewards (i.e., classroom party and t-shirts) for F/V consumption in the cafeteria	Digital imagery	Selection: There was a 16% increase in the proportion of students selecting a vegetable (*p* < 0.01). Consumption: The intervention was associated with a significant increase in the proportion of students consuming all their fruits (11% increase; *p* < 0.01) and all their vegetables (8.7% increase; *p* < 0.01). There was also a decrease in the percent of students not consuming any of the fruits on their trays (16.0% decrease; *p* < 0.001).	High
Mazzeo et al. 2017 [71]	Mid-Atlantic region; 2 elementary schools (2087 trays from students in grades 1–3)	QE: Pre/post (with comparison group)	2014–15	(1) Choice Architecture: Students were rewarded for eating F/V (i.e., sticker and praise) (2) Taste Tests	Visual estimation	Selection: Not measured Consumption: The intervention was associated with reduced F/V waste.	High
Morrill et al. 2016 [72]	UT; 6 elementary schools (2292 students in grades	QE: Pre/post (with comparison group)	2011	(1) Choice Architecture: Students were rewarded for eating F/V (i.e., toys or praise from teachers). Adults provided role modeling. (2) Taste Tests (3) Other: Videos and letters	Visual estimation	Selection: Not measured Consumption: The intervention was associated with increased F/V consumption. At 6 months follow-up (after the intervention ended), only the intervention arm with prizes (plus nutrition education and taste tests) was associated with sustained increased consumption.	Low
Perry et al. 2004 [48]	St. Paul, MN; 26 elementary schools (1668 students in grades 1 and 3)	RCT	2000–2002	(1) Choice Architecture: Verbal prompts by food service staff encouraging consumption; improved attractiveness of F/V (e.g., placing them in small cups; arranging by color); posters and characters (life size fruit and vegetables); and rewards for eating F/V (classroom receives frozen fruit yogurt if enough students ate 3 servings at lunch) (2) Choices: The quantity of F/Vs was increased (3) Taste Tests	Visual estimation	Selection: Not measured Consumption: The intervention was associated with significant increases in total servings of F/V (excluding potatoes and juice).	Low
Wengreen et al. 2013 [81]	UT; 1 elementary school (253 students in grades 1–5)	QE: Pre/post (no comparison group)	2010-11	(1) Choice Architecture: Students received rewards (e.g., pencil eraser, pedometer) for trying F/V (2) Other: Videos and letters were read by the teacher	Digital imagery	Selection: Not measured Consumption: The intervention was associated with significant increases in fruit and vegetable consumption.	High
Choice Architecture: Visual Appeal, verbal prompts, and re-ordering the lunch line							
Adams et al. 2016 [88]	Phoenix, AZ; 2 school districts (3 middle schools per district [students total])	QE: Post-only (with comparison group)	2013	Choice Architecture: The placement of the salad bar was either on the serving line or after the serving line	Weighed plate waste	Selection: Students selected 5.4 times more fresh fruits and vegetables by weight (95% CI, 4.0–7.2) when the salad bar was on the serving line (vs. after the serving line). Consumption: Students consumed 4.83 times more F/V (95% CI 3.40 to 6.81) when the salad bar was on the serving line (vs. after the serving line).	Low
Ang et al. 2019 [51]	New York City, NY; 14 elementary schools [877 trays collected from students in grade 2–3)	QE: Post-only (with comparison group)	2015–16	(1) Choice Architecture: Vegetables were pre-plated (vs. optional), and were placed first in line (2) Pre-sliced Fruit (3) Recess before lunch (4) Choices: multiple (2+) fruit and vegetable options were provided (5) Lunch Duration	Visual estimation	Selection: Not measured Consumption: Pre-plating vegetables (vs. optional for the student to select a vegetable) was associated with a small increase in consumption (0.02 cups; *p* < 0.001). Positioning vegetables first on the serving line was not associated with vegetable consumption. Among students who selected fruit, pre-sliced fruit was associated with greater consumption (0.23 cups more *p* = 0.02) than whole fruit. Recess before lunch was associated with a small increase in fruit consumption (0.08 cups; *p* < 0.001) and vegetable consumption (0.007 cups; *p* = 0.04). Multiple fruit options and attractive serving bowls were not associated with fruit consumption. Lunch duration was not associated with consumption (although less than 15% of measurements had lunch durations of ≥20 min).	Low
Blondin et al. 2018 [93]	N.S.; 6 elementary schools (students in grades 3–4)	Cross-sectional	2015	(1) Choice Architecture: Teachers encouraged students to select milk (2) Other: Distractions (i.e., other activities while eating including working, listening to the teacher, and/or socializing) were assessed and students were offered juice with breakfast on approximately half of the days	Weighed plate waste	Selection: When juice was offered with breakfast, the percent of students selecting milk decreased. Consumption: Offering juice at breakfast was associated with a 12% increase in milk waste (*p* < 0.001). Teacher encouragement to select milk was associated with a 9% increase in milk waste (*p* = 0.009). Student engagement in other activities during breakfast was associated with a 10% decrease in milk waste (*p* < 0.001).	Low
Cohen et al. 2015 [57]	MA; 14 elementary and middle schools (2638 students in grades 3–8)	RCT	2011–12	(1) Choice architecture: Vegetables were offered at the beginning of the lunch line; fruits were placed in attractive containers; fruits were placed next to the cash register; signage added that promoted F/V; white milk was placed in front of chocolate milk (2) Palatability: A professional chef trained cafeteria staff to prepare healthier school lunches	Weighed plate waste	Selection: Both the choice architecture and chef (i.e., palatability) intervention were associated with increased fruit and vegetable selection. There was no impact on white milk selection. Consumption: Only the chef intervention was associated with increased consumption of fruits and vegetables. There was no impact on white milk consumption.	Low
Goto et al. 2013 [90]	CA; 3 elementary schools (677 students in grades 1–6)	RCT	2011	Choice Architecture: The quantity of white milk was increased compared with the quantity of chocolate milk. Some schools requested to decrease the visibility of chocolate milk from the lunch line.	Weighed plate waste	Selection: When the visibility of chocolate milk was decreased, there was an 18% percentage-point increase in white milk selection. Changing the quantity of white milk available was not associated with changes in selection. Consumption: The interventions were not associated with differences in white milk consumption.	High
Greene et al. 2017 [49]	NY; 7 middle schools (8502 trays from students in grades 5–8)	RCT	2014	(1) Choice Architecture: Fruits were placed first on the lunch line and were in attractive bowls with descriptive names. Promotional materials (e.g., fruit facts) were posted in the cafeteria (2) Choices: Multiple (2) fruit and vegetable options were provided (3) Pre-sliced Fruit	Visual estimation	Selection: The intervention was associated with a 36% increase in fruit selection (*p* < 0.001), but no significant changes in vegetable or milk selection. Consumption: The intervention was associated with a 23% increase in fruit consumption (*p* < 0.001). There was no association with vegetable or milk consumption.	Low
Gustafson et al. 2017 [87]	Kearney, N; 4 elementary schools (1614 trays from students in grades K-5)	RCT	2014–15	(1) Choice architecture: Posters marketing vegetables were mounted above the salad bar (2) Other: Students participated in the design of the posters	Digital imagery	Selection: The intervention arm with both student participation in the poster design and the presence of marketing was associated with an increase in the selection of vegetables. Consumption: The intervention arm with both student participation in the poster design and the presence of marketing was associated with a significant increase in vegetable consumption compared with the control group.	High
Hamdi et al. 2020 [63]	IL; 3 elementary schools (1255 trays from students in grades K-8)	QE: Pre/post (no comparison group)	2018–19	(1) Choice Architecture: Cafeteria decorations and creative names were introduced (2) Taste Tests (3) Palatability: A flavor station with spices and seasonings on a table in the cafeteria was added	Weighed plate waste	Selection: Selection was measured in only one of the participating schools. The odds of selecting a vegetable (i.e., broccoli) increased when students were exposed to taste tests. The odds of selecting fruit increased when all intervention components were implemented simultaneously. Consumption: The intervention components yielded inconsistent, but generally positive consumption results across the schools, particularly for fruits.	Low
Hanks et al. 2012 [91]	Corning, NY; 1 high school (1084 trays from students)	QE: Pre/post (no comparison group)	2011	(1) Choice Architecture: A convenience line (i.e., faster lunch line) was added with only healthier options	Weighed plate waste	Selection: The intervention was associated with an 18% increase in healthier food selection (*p* < 0.001). Consumption: There was no significant change in the consumption of healthy foods, but there was a significant decrease in consumption of less healthy foods (27.9% decrease; *p* < 0.001).	High
Hanks et al. 2013 [89]	NY; Two Middle/High schools (3762 trays from students in grades 7–12).	QE: Pre/post (no comparison group)	2011	Choice Architecture: The following components were included: a convenience line with healthier options; placing healthier foods first in line; adding attractive bowls and descriptive names; and providing verbal prompts to select healthy options	Visual estimation	Selection: Students were 13.4% more likely to take a fruit (*p* = 0.01) and 23% more likely to take a vegetable (*p* < 0.001) post-implementation. Consumption: Choice architecture was associated with an 18% increase in fruit consumption (*p* = 0.004) and 25% increase in vegetable consumption (*p* < 0.001).	High
Koch et al. 2020 [92]	New York City, NY; 7 high schools	QE: Pre/post (no comparison group)	2017 to 2018	Choice Architecture: The intervention included a more open lunch line, comfortable seating options, wall art, and promotional signage	Digital imagery	Selection: Fruit and vegetable selection decreased (statistical significance not assessed). Consumption: After a year of exposure to the intervention, there were no changes in vegetable consumption and significant decreases in consumption of fruits and grains.	High
Quinn et al. 2018 [70]	King County, WA; 11 middle and high schools (2309 trays)	QE: Pre/post (with comparison group)	2013–14	(1) Pre-sliced Fruit (2) Choice Architecture: Changes included attractive containers, creative names, signage, food placement (e.g., multiple locations and/or at eye-level), and verbal prompts by cafeteria staff	Visual estimation	Selection: A greater proportion of students selected fruit in the intervention schools compared with control schools. There was no significant change in vegetable selection. Consumption: Pre-sliced fruit and choice architecture were not associated with significant differences in the quantities of fruits, vegetables, or milk consumed.	High
Reicks et al. 2012 [96]	Richfield, MN; 1 elementary school (students in grades K-5)	QE: Pre/post (no comparison group)	2011	Choice Architecture: Photographs of vegetables were placed on lunch trays	Aggregate plate waste	Selection: The intervention was associated with an 8.5% percentage point increase in green beans (*p* < 0.001) and a 25.2% percentage point increase in carrot selection (*p* < 0.001). Consumption: Among students who selected vegetables, the intervention was associated with a decrease in carrot consumption (27g vs. 31g; *p* < 0.001) and no impact on green bean consumption.	Very High
Schwartz 2007 [94]	CT; 2 elementary schools (students in grades 1–4)	QE: Post-only (with comparison group)	2005	Choice Architecture: Staff provided verbal prompts on the lunch lune encouraging fruit selection	Visual estimation	Selection: The intervention was associated with significantly greater odds of selecting fruit. Consumption: Among students who selected a fruit, there were no differences in fruit consumption.	High
Thompson et al. 2017 [69]	Hennepin County, MN; 2 elementary schools (373 students in grades K-4)	QE: Pre/post (no comparison group)	2013	(1) Choice Architecture: Changes included enhanced displays for F/V; attractive labels for F/V; and placement of F/V at the beginning of the lunch line and at the cash register (2) Pre-sliced Fruit: Sliced apples were compared with whole apples	Weighed plate waste	Selection: The intervention was associated with a significant increase in the percentage of students selecting a fruit serving (95.1% vs. 98.1%; *p* = 0.02). There was no significant change in vegetable selection. Consumption: The intervention was not associated with fruit or vegetable consumption.	Low
Wansink et al. 2015 [95]	Lansing, NY; 1 high school (554 trays from students in grades 9–12)	QE: Pre/post (no comparison group)	2012	Choice Architecture: Salad greens grown by one classroom were added to the school salads, and posters and announcements were introduced to promote this addition	Visual estimation	Selection: The intervention was associated with an increase in salad selection from 2% to 10% (*p* < 0.001). Consumption: The intervention was associated with a decrease in salad consumption (94% to 67% of a serving consumed; *p* = 0.007).	High
Choice Architecture: Portion Sizes or Modifying When Students Have Access to Meal Components							
Elsbernd et al. 2016 [100]	Richfield, MN; 1 elementary school (~500 students in grades K-5)	QE: Pre/post (no comparison group)	N.S.	Choice Architecture: Students were offered vegetables (red pepper) in the hallway outside the cafeteria while waiting on the lunch line. Staff provided verbal prompts to eat the peppers while waiting on the lunch line.	Visual estimation	Selection: The selection of red pepper increased from 8% to 65% (statistical significance not assessed). Consumption: The intervention was associated with an increase in overall vegetable consumption.	High
Miller et al. 2015 [99]	Richfield, MN; 1 elementary school (~680 students in grades K-5)	QE: Pre/post (no comparison group)	2011	Choice Architecture: There was a 50% increase in portion sizes for F/V	Weighed plate waste	Selection: Larger portion sizes was associated with a significant increase in the proportion of students selecting oranges and a decrease in the proportion of students selecting applesauce. Consumption: Increasing the portion sizes for F/V was associated with increased consumption (range 13–42g increase) among those who selected the meal component.	Low
Ramsay et al. 2013 [97]	N.S.; 1 Kinder Center (elementary school with only kindergarten)	QE: Pre/post (no comparison group)	2010	Choice Architecture: An increase in the portion size of the entrée (i.e., the number of chicken nuggets offered)	Weighed plate waste	Selection: Students selected more chicken nuggets when they were able to choose larger serving sizes. Consumption: Larger portion sizes of chicken nuggets were associated with greater consumption.	High
Redden et al. 2015 [101]	Richfield, MN; 1 elementary school (1435 trays [study 1] and 2632 trays [study 2] from students in grades K-5)	QE: Pre/post (no comparison group)	N.S.	Choice Architecture: In Study 1, vegetables (mini carrots) were available on a table while students waited in line for food. In Study 2, vegetables (broccoli) were handed to students while they waited in line for food.	Aggregate plate waste	Selection: No significant associations Consumption: In Study 1, offering mini carrots to students while they waited in the lunch line was associated with an overall increase in carrot consumption at lunch compared with a control day (12.7g vs. 2.4g; *p* < 0.001). In Study 2, with a longer exposure to the intervention, offering broccoli was associated with increased consumption that persisted over time.	Very High
Zellner et al. 2016 [102]	Philadelphia, PA; 1 elementary school (47 trays from students in grades 3–4)	QE: Pre/post (no comparison group)	N.S.	Choice Architecture: Fruit was served later in the meal versus at the same time as the rest of the school lunch	Visual estimation	Selection: Not measured Consumption: Delaying when fruit was served was associated with greater kale consumption (*p* = 0.0017) compared with when fruit was served at the same time as the rest of the meal.	Very High

F/V: fruits and vegetables; QE: Quasi-Experimental; RCT: Randomized controlled trial. ^1^ Study designs were defined as: (1) Cross-sectional- Observational study with the exposure and outcome measured simultaneously (no comparison group); (2) Quasi-Experimental (QE): Post Only—Intervention study with a comparison group and data collected post-implementation (no baseline measurements); (3) Quasi-Experimental (QE): Pre/Post—Intervention studies with pre-implementation (i.e., baseline) and post-implementation measurements, with or without a comparison group (non-random allocation of intervention/comparison groups); (4) Randomized Controlled Trial (RCT)—Intervention study with random allocation to intervention or control status and both pre-implementation (baseline) and post-implementation measurements. ^2^ Risk of Bias was based on adapted Newcastle–Ottawa Scales (NOS) for cross-sectional and cohort studies (Supplementary Materials Table S1).

**Table 3:** Characteristics and outcomes of studies examining initiatives and interventions targeting nutrition education included in the systematic review.

Author, Year	Location; Participant Characteristics	Study Design ^1^	Year(s)	Exposure(s)	Outcome Measure(s)	Results	**Risk of Bias^2^**
Nutrition Education							
Auld et al. 1998 [105]	Denver, CO; 10 elementary schools (3 intervention and 3 comparison) [~850 students in grades k-5]	QE: Pre/post (with comparison group)	1995–96 to 1996–97	(1) Nutrition Education: 24 nutrition lessons were taught by special resource teachers (2) Other: Teacher training and parent education (newsletters) provided	Visual estimation	Selection: Not measured Consumption: The intervention was associated with an increase in F/V consumption by 0.4 serving (*p* < 0.001).	Low
Auld et al. 1999 [104]	Denver, CO; 4 elementary schools (2 intervention and 2 control [~760 students total in grades 2–4])	QE: Pre/post (with comparison group)	1997–98	Nutrition Education: 16 nutrition lessons were taught alternatively by teachers and special resource teachers	Visual estimation	Selection: Not measured Consumption: Nutrition education was associated with an increase in F/V consumption by 0.36 servings (*p* < 0.001).	Low
Burgess-Champoux et al. 2008 [106]	Minneapolis metropolitan area, MN: 2 elementary schools (150 students in grades 4 and 5)	QE: Pre/post (with comparison group)	2005	(1) Nutrition Education: Five lessons that focused on whole grains were implemented by trained research assistants (2) Other: There was a family component (newsletters, supermarket and bakery tours, and an event at a milling museum). To increase the availability of whole grains in the cafeteria, there was culinary training on whole grains for cafeteria staff focused on menu planning, procurement, quality control and staff taste tests.	Visual estimation	Selection: Not measured Consumption: In the intervention school, whole grain consumption increased by 1 serving (*p* < 0.0001) and refined grain consumption decreased by 1 serving (*p* < 0.001) compared with the control school.	High
Epstein-Solfield et al. 2018 [111]	WA; 1 elementary school (149 students in grades 3 and 5)	QE: Pre/post (no comparison group)	2017	Nutrition Education: Lessons were provided for 8 weeks (20 min per session) focused on the benefits of consuming F/V	Visual estimation	Selection: Not measured Consumption: The intervention was not associated with differences in F/V consumption.	High
Head 1974 [107]	NC; 4 elementary, 4 middle, and 2 high schools (students in grade 5, 7, and 10)	QE: Pre/post (with comparison group)	N.S.	Nutrition Education: Lessons were provided on basic nutrition, dietary patterns, and food composition	Weighed plate waste	Selection: Not measured Consumption: There was a significant decrease in plate waste with nutrition education.	Low
Ishdorj et al. 2013 [110]	Nationally representative sample (SNDA-III); 256 schools (2096 students)	Cross-sectional	2004–05	(1) Nutrition Education: Availability of nutrition education lessons for every grade in the school (2) Policy: restrictions on competitive foods (e.g., limited à la carte sales, no stores or snack bars selling competitive foods, and fundraisers), French fries, dessert, and whole/ 2% milk; and increases in the amount of fresh F/V available daily at lunch	24 h recall	Selection: Not measured Consumption: Policies that place restrictions on the sales of competitive foods were associated with greater fruit consumption. Policies that restrict desserts were associated with greater vegetable consumption. Policies that limited French fries were associated with lower fruit consumption. Limiting whole and 2% milk was associated with greater fruit and vegetable consumption. Nutrition education and policies that increase the amount of fresh fruit and vegetable available daily at lunch policies were not associated with consumption of fruits or vegetables.	High
Jones et al. 2015 [109]	SC; 15 elementary and 3 middle schools (students in grades K-8)	QE: Post-only (with comparison group)	2011	(1) Nutrition Education: Nutrition and agriculture content was integrated into classroom curriculum (2) Other: Schools incorporated farm to school activities, including a school garden, field trips to farms, cooking demonstrations and providing local items on the cafeteria menu	Digital imagery	Selection: Not measured Consumption: Students in the intervention schools consumed on average less fruit than students in control schools. There was no significant association with vegetable consumption.	High
Larson et al. 2018 [112]	Southwest region; 2 elementary schools (159 students in grades 4–5)	QE: Pre/post (with comparison group	N.S.	Nutrition Education: Fruit and vegetable consumption was promoted via cartoon characters, posters, and goal setting	Visual estimation	Selection: Not measured Consumption: The intervention was not associated with differences in F/V consumption.	High
Prescott et al. 2019 [108]	CO; 2 middle schools (1596 trays from students in grade 6–8)	QE: Pre/post (with comparison group)	2017–18	Nutrition education: The 6th grade curriculum focused on sustainable food systems. 6th grade students created posters to educate the 7–8th grade students	Digital imagery	Selection: No significant associations Consumption: During the intervention, students in intervention school increased their vegetable consumption (due to significantly lower consumption rates at baseline, the intervention eliminated the difference versus control). At 5 months follow-up, the intervention students wasted significantly less salad bar vegetables compared with the control students (24g vs. 50g; *p* = 0.029).	High
Serebrennikov et al. 2020 [113]	Midwestern Region; 3 elementary schools (98 students in grade 2)	RCT	2016	Nutrition Education: A curriculum related to knowledge and preferences for F/V was implemented bi-weekly for 6 weeks	Digital imagery	Selection: No significant associations Consumption: Nutrition education was not associated with significant differences in fruits or vegetables consumed.	Low
Sharma et al. 2019 [103]	Houston/Dallas, TX; 3 elementary schools (115 students in grades 4–5)	QE: Pre/post (with comparison group)	2017-18	(1) Nutrition Education: A 16-week program was provided (2) Other: A parent education component included sending home recipes with demonstrations and fresh fruit from local pantries	Weighed plate waste	Selection: There was no change in selection among intervention schools but there was a decrease in the comparison schools, which resulted in a significant difference in selection. Consumption: The intervention was associated with significant decreases in F/V waste at lunch (*p* < 0.001).	Low
Multi-Component Nutrition Education with Choice and/or Taste Test Components							
Alaimo et al. 2015 [73]	Grand Rapids, MI; 6 elementary schools (4 intervention and 2 control [815 students in grades 3-5])	QE: Pre/post (with comparison group)	2009–10 to 2010–11	(1) Taste Tests: Provided in the cafeteria and classrooms (2) Nutrition Education: Nutrition education classes and posters (3) Other: Healthy eating coaching by teachers, parent education	Digital imagery	Selection: Not measured Consumption: The multi-component intervention was associated with significant increases in fruit consumption. No differences in consumption of vegetables, milk, grains, or protein were observed.	Low
Blakeway et al. 1978 [75]	Little Rock, AR; 16 elementary schools (5000 students in grades 1–3)	RCT	N.S.	(1) Taste Tests: Provided in the classroom with nutrition education (2) Nutrition Education: A nutrition coordinator implemented 10 classroom lessons focused on recognizing and identifying foods in different forms	Aggregate plate waste	Selection: Not measured Consumption: Students in intervention schools consumed greater amounts of whole wheat rolls (grades 2 and 3 only) and cottage cheese (grades 1 and 2 only) compared to the comparison group. Sweet potato custard consumption increased in both the intervention and control group. No other significant differences were observed.	High
Blom-Hoffman et al. 2004 [85]	Northeast Region; 1 elementary school (students in grades K-1)	RCT	N.S.	(1) Choice Architecture: Students received verbal praise and rewards (i.e., stickers) for consuming F/V (2) Nutrition Education: 10 lessons with 5-A-Day information was implemented by the classroom teacher and a school psychology graduate student (3) Other: Parent component (newsletter and cookbook) included	Visual estimation	Selection: Not measured Consumption: The intervention was not associated with differences in vegetable consumption.	High
Bontranger Yoder et al. 2014 [77]	WI; 9 elementary schools (1117 students in grades 3–5)	QE: Pre/post (no comparison group)	2010-11	(1) Nutrition Education: A farm to school gardening curriculum was available in some of the participating schools (2) Taste tests: Available in some of the participating schools (3) Other: Farm to school activities were introduced (e.g., a school garden, field trips to farms, and local items on the menu) in some of the participating schools	Digital imagery	Selection: Not measured Consumption: The intervention was not associated with differences in F/V consumption, although the farm to school components were inconsistently implemented across the participating schools.	Low
Bontranger Yoder et al. 2015 [78]	WI; 11 elementary schools (7117 trays from students in grades 3–5)	Cross-sectional and Pre/post (no comparison group) * * For Policy only	2010 to 2013	(1) Nutrition Education: A farm to school gardening curriculum was available in some of the participating schools (2) Taste tests: Available in some of the participating schools. (3) Policy: HHFKA (4) Other: Farm to school activities were introduced (e.g., a school garden, field trips to farms, and local items on the menu) in some of the participating schools	Digital imagery	Selection: Not measured Consumption: The intervention components (i.e., nutrition education, taste tests, and other activities) were not associated with differences in F/V consumption. There was no change in consumption before or after implementation of the HHFKA.	High
Georgiou (1998 [Gov’t Report]) [74]	OR; 1 elementary school (40 students in grade 3)	QE: Pre/post (with comparison group)	1997	(1) Nutrition Education: Lessons included the Food Guide Pyramid, healthy meal planning, and how foods grow (2) Taste Tests: Provided in the classroom with nutrition education	Weighed plate waste	Selection: No significant associations Consumption: The intervention was associated with an increase in consumption of calories from fruits (28 kcal; *p* < 0.01). No significant differences in vegetable and grain consumption were observed.	High
Liquori et al. 1998 [50]	New York City, NY; 2 elementary schools (590 students in grades K-6).	QE: Pre/post (with comparison group)	1995-96	(1) Choices: The number of vegetable and whole grain options available was increased (2) Nutrition Education: 10 food and environment lessons and/or 10 cooking lessons were provided (3) Other: Students took field trips to a local community garden. There was parent outreach (newsletter, recipes, workshops)	Visual estimation	Selection: Not measured Consumption: The intervention arm with cooking lessons was associated with increased consumption of vegetables and whole grains among younger students (*p* < 0.01). No association was observed among older children exposed to the cooking intervention. The nutrition education (food environment) intervention was not associated with consumption.	High
Reynolds et al. 2000 [76]	AL; 28 elementary schools (425 students in grade 4)	QE: Pre/post (with comparison group)	1994 to 1996	(1) Nutrition Education: A 14 lesson curriculum related to F/V was provided and posters were added to the cafeteria (2) Taste Tests (3) Other: Parent education was provided, including recipes, activities, and information about F/V	Visual estimation	Selection: Not measured Consumption: The intervention was not associated with significant differences in fruits or vegetables consumed.	Low
Taylor et al. 2018 [52]	CA; 2 elementary schools (112 students in grade 4)	QE: Pre/post (with comparison group)	2012–13	(1) Choices: Salad bars were added to increase F/V choices (2) Nutrition Education: A garden enhanced nutrition curriculum and cooking demonstrations were provided (3) Other: Parent newsletter, home activities	Digital imagery	Selection: No significant associations Consumption: The intervention was associated with a significant increase in vegetable consumption. There was no association with fruit consumption.	High
Young et al. 2013 [47]	N.S.; 1 middle school (3810 trays from students in grades 6–8)	Cross-Sectional	2011–12	Policy: A new wellness policy required schools to implement the practices below: (1) Choices: Different fruits and vegetables were served each day of the week (2) Nutrition Education: There was an increase in student contact hours for health and physical education	Visual estimation	Selection: Not measured Consumption: After exposure to the wellness policy for over a semester, students consumed significantly more fruits and cooked vegetables.	High

F/V: fruits and vegetables; QE: Quasi-Experimental; RCT: Randomized controlled trial. ^1^ Study designs were defined as: (1) Cross-sectional- Observational study with the exposure and outcome measured simultaneously (no comparison group); (2) Quasi-Experimental (QE): Post Only Intervention study with a comparison group and data collected post-implementation (no baseline measurements); (3) Quasi-Experimental (QE): Pre/Post—Intervention studies with pre-implementation (i.e., baseline) and post-implementation measurements, with or without a comparison group (non-random allocation of intervention/comparison groups); (4) Randomized Controlled Trial (RCT)—Intervention study with random allocation to intervention or control status and both pre-implementation (baseline) and post-implementation measurements. ^2^ Risk of Bias was based on adapted Newcastle–Ottawa Scales (NOS) for cross-sectional and cohort studies (Supplementary Materials Table S1).

**Table 4:** Characteristics and outcomes of studies examining initiatives and interventions targeting other cafeteria environment-level factors included in the systematic review.

Author, Year	Location; Participant Characteristics	Study Design ^1^	Year(s)	Exposure(s)	Outcome Measure(s)	Results	Risk of Bias ^2^
**School Lunch Duration**							
Ang et al. 2019 [51]	New York City, NY; 14 elementary schools (877 trays collected from students in grade 2–3)	QE: Post-only (with comparison group)	2015–16	(1) Choice Architecture: Vegetables were pre-plated (vs. optional), and were placed first in line (2) Pre-sliced Fruit (3) Recess before lunch (4) Choices: multiple (2+) fruit and vegetable options were provided (5) Lunch Duration	Visual estimation	Selection: Not measured Consumption: Pre-plating vegetables (vs. optional for the student to select a vegetable) was associated with a small increase in consumption (0.02 cups; *p* < 0.001). Positioning vegetables first on the serving line was not associated with vegetable consumption Among students who selected fruit, pre-sliced fruit was associated with greater consumption (0.23 cups more *p* = 0.02) than whole fruit. Recess before lunch was associated with a small increase in fruit consumption (0.08 cups; *p* < 0.001) and vegetable consumption (0.007 cups; *p* = 0.04). Multiple fruit options and attractive serving bowls were not associated with fruit consumption. Lunch duration was not associated with consumption (although less than 15% of measurements had lunch durations of ≥20 min).	Low
Bergman et al. 2004 A [114]	WA; 2 elementary schools (1877 trays from students in grades 3–5)	QE: Post-only (with comparison group)	N.S.	Lunch Duration: The times varied from 20–30 min	Weighed plate waste	Selection: Not measured Consumption: Longer lunch periods were associated with significantly greater school meal consumption (72.8% vs. 56.5% consumed; *p* < 0.0001).	High
Cohen et al. 2016 [19]	MA; 6 elementary and middle schools (1001 students in grades 3–8)	QE: Post-only (with comparison group)	2011–12	Lunch Duration: The times varied from 20–30 min (the amount of seated time in the cafeteria was calculated)	Weighed plate waste	Selection: Fruit selection was lower when students had less time to eat (46.9% vs. 57.3%; *p* < 0.0001). Consumption: A shorter lunch period (less than 20 min of seated time) was associated with a decreased consumption of entrées (12.8% reduction; *p* < 0.0001), milk (10.3% reduction; *p*<0.0001), and vegetables (11.8% reduction, *p*<0.0001).	Low
Gross et al. 2018 [115]	New York City, NY; 10 elementary schools (382 students ages 6–8 years)	QE: Post-only (with comparison group)	2013	(1) Lunch Duration: The times were ≥30 min vs. <30 min (2) Other: Noise levels and crowding were assessed	Digital imagery	Selection: On average, 74% of students selected a fruit, 69% selected a vegetable, and 73% selected a whole grain (statistical significance not assessed). Consumption: A longer lunch duration (≥30 min) was associated with higher consumption of fruits (odds ratio [OR] = 2.0; *p* = 0.02) and whole grains (OR = 2.1; *p* <0.05). Quieter cafeterias were associated with eating more vegetables (OR = 3.9; *p* < 0.001) and whole grains (OR= 2.7; *p* < 0.001). Less crowding was associated with eating more fruit (OR = 2.3; *p* = 0.04) and whole grains (OR = 3.3; *p* < 0.001).	Low
Recess Before Lunch							
Ang et al. 2019 [51]	New York City, NY; 14 elementary schools [877 trays collected from students in grade 2–3)	QE: Post-only (with comparison group)	2015–16	(1) Choice Architecture: Vegetables were pre-plated (vs. optional), and were placed first in line (2) Pre-sliced Fruit (3) Recess before lunch (4) Choices: multiple (2+) fruit and vegetable options were provided (5) Lunch Duration	Visual estimation	Selection: Not measured Consumption: Pre-plating vegetables (vs. optional for the student to select a vegetable) was associated with a small increase in consumption (0.02 cups; *p* < 0.001). Positioning vegetables first on the serving line was not associated with vegetable consumption Among students who selected fruit, pre-sliced fruit was associated with greater consumption (0.23 cups more *p* = 0.02) than whole fruit.. Recess before lunch was associated with a small increase in fruit consumption (0.08 cups; *p* < 0.001) and vegetable consumption (0.007 cups; *p* = 0.04). Multiple fruit options and attractive serving bowls were not associated with fruit consumption. Lunch duration was not associated with consumption (although less than 15% of measurements had lunch durations of ≥20 min).	Low
Bergman et al. 2004 B [116]	W; 2 elementary schools (2008 trays from students grades 3–5)	QE: Post-only (with comparison group)	N.S.	Recess before lunch	Weighed plate waste	Selection: Not measured Consumption: Recess before lunch was associated with significantly greater school meal consumption (72.8% vs. 59.9% consumed; *p* < 0.0001).	High
Chapman et al. 2017 [117]	New Orleans, LA; 8 elementary schools (20,183 trays from students in grades 4 and 5)	QE: Post-only (with comparison group)	2014	(1) Recess before lunch (2) Other: Timing of lunch varied (early, midday, or late)	Weighed plate waste	Selection: Not measured Consumption: Recess before lunch was associated with a 5.1% increase in fruit consumption (*p* = 0.009). There was no association between the timing of recess and consumption of the entrée, vegetable, or milk. Students who had a very early lunch consumed 5.8% less of their entrées (*p* < 0.001) and 4.5% less of their milk (*p* = 0.047) compared with students who had lunch at a traditional lunch hour. Additionally, students who had a very late lunch consumed 13.8% less of their entrées (*p* < 0.001) and 15.9% less of their fruit (*p* < 0.001).	Low
Fenton et al. 2015 [123]	CA; 31 elementary schools (2167 students in grades 4–5).	QE: Post-only (with comparison group)	2011–12	Recess before lunch	24 hour recalls (diary assisted)	Selection: Not measured Consumption: Recess before lunch was not associated with differences in FV consumption at lunch.	Low
Getlinger et al. 1996 [118]	Rockfort, IL; 1 elementary school (67 students in grades 1–3)	QE: Pre/post (no comparison group)	1995	Recess before lunch	Weighed plate waste	Selection: Not measured Consumption: Recess before lunch was associated with significant reductions in food waste for meat/meat alternatives, vegetables, and milk. There were no significant differences observed for fruits or grains.	High
Hunsberger et al. 2014 [21]	Madras, OR; 1 elementary school (261 students in grades K-2)	QE: Post-only (with comparison group)	2009–10	Recess before lunch	Weighed plate waste	Selection: Not measured Consumption: Students with recess before lunch were roughly 20% more likely to drink an entire carton of milk (42% vs. 25%; *p* < 0.001) and consumed on average 1.3 oz more milk compared with students who had recess after lunch. There were no significant differences in consumption of entrées, vegetables, or fruits.	High
McLoughlin et al. 2019 [120]	IL; 2 elementary schools (103 students in grades 4–5)	QE: Post-only (with comparison group)	2016	Recess before lunch	Weighed plate waste	Selection: Overall, 57% of students selected fruits, 26% selected vegetables, 68% selected entrées and 64% selected milk (statistical significance not assessed). Consumption: Recess before lunch was associated with on average greater milk consumption. There was no association between recess before lunch and the amount of entrée, fruit, or vegetable consumed.	High
Price et al. 2015 [122]	Orem, UT; 7 elementary schools (22,939 trays from students in grades 1–6)	QE: Pre/post (with comparison group)	2010–11 to 2011–12	Recess before lunch	Visual estimation	Selection: Not measured Consumption: Recess before lunch was associated with a 0.16 serving increase in fruit and vegetable consumption (*p* < 0.01).	Low
Strohbehn et al. 2016 [119]	Midwestern Region; 3 elementary school (students in grade 3)	QE: Pre/post (no comparison group)	2012	Recess before lunch	Digital imagery and weighed plate waste	Selection: Not measured Consumption: Recess before lunch was associated with inconsistent findings; while the average waste was reduced for grains, meat/meat alternatives, and fruits, the average waste increased for vegetables.	High
Tanaka et al. 2005 [121]	Oahu, HI; 1 elementary school (students in 6th grade)	QE: Pre/post (no comparison group)	2004	Recess before lunch	Weighed plate waste	Selection: Not measured Consumption: Recess before lunch was not associated with consumption of F/V, milk, or other school meal components.	High

F/V: fruits and vegetables; QE: Quasi-Experimental; RCT: Randomized controlled trial. ^1^ Study designs were defined as: (1) Cross-sectional- Observational study with the exposure and outcome measured simultaneously (no comparison group); (2) Quasi-Experimental (QE): Post Only—Intervention study with a comparison group and data collected post-implementation (no baseline measurements); (3) Quasi-Experimental (QE): Pre/Post—Intervention studies with pre-implementation (i.e., baseline) and post-implementation measurements, with or without a comparison group (non-random allocation of intervention/comparison groups); (4) Randomized Controlled Trial (RCT)—Intervention study with random allocation to intervention or control status and both pre-implementation (baseline) and post-implementation measurements. ^2^ Risk of Bias was based on adapted Newcastle–Ottawa Scales (NOS) for cross-sectional and cohort studies (Supplementary Materials Table S1).

**Table 5:** Characteristics and outcomes of studies examining policy-level factors included in the systematic review.

**Author, Year**	**Location; Participant Characteristics**	**Study Design ^1^**	**Year(s)**	**Exposure(s)**	**Outcome Measure(s)**	**Results**	**Risk of Bias ^2^**
**Healthy, Hunger-Free Kids Act (HFFKA)**							
Amin et al. 2015 [124]	Northeast Region; 2 elementary schools (1442 trays from students in grades 3-5)	QE: Pre/post (no comparison group)	Spring 2012 and Spring 2013	Policy: HHFKA	Digital imagery, weighed plate waste, and direct observation	Selection: The policy was associated with a significant increase in FV selection (97.5% vs. 84.3%; *p* < 0.001). Consumption: The policy was associated with slightly lower FV consumption (0.51 cups vs. 0.45 cups, *p <* 0.01)	High
Bontranger Yoder et al. 2015 [78]	WI; 11 elementary schools (7117 trays from students in grades 3–5)	Cross-sectional and Pre/post (no comparison group) * * For Policy only	2010 to 2013	(1) Nutrition Education: A farm to school gardening curriculum was available in some of the participating schools (2) Taste tests: Available in some of the participating schools (3) Policy: HHFKA (4) Other: Farm to school activities were introduced (e.g., a school garden, field trips to farms, and local items on the menu) in some of the participating schools	Digital imagery	Selection: Not measured Consumption: The intervention components (i.e., nutrition education, taste tests, and other activities) were not associated with differences in F/V consumption. There was no change in consumption before or after implementation of the HHFKA.	High
Cohen et al. 2014 [24]	MA; 4 elementary/K-8 schools (1030 students in grades 3–8)	QE: Pre/post (no comparison group)	Fall 2011 and Fall 2012	Policy: HHFKA + removal of chocolate milk	Weighed plate waste	Selection: The HHFKA was associated with a 23% increase in the percent of students selecting a fruit (*p* < 0.0001). There was no association with vegetable selection. Milk selection decreased by 24.7% (*p* < 0.0001) when chocolate milk was removed. Consumption: The HHFKA was associated with increased entrée consumption (15.6% increase; *p* < 0.0001) and vegetable consumption (16.2% increase; *p* < 0.0001). Milk consumption decreased by 10% when chocolate milk was removed (*p* < 0.0001). There was no impact on fruit consumption.	Low
Cullen et al. 2015B [26]	TX; 8 elementary schools (1045 trays from students in grades	QE: Pre/post (no comparison group)	Spring 2011 & Spring 2013	Policy: HHFKA	Visual estimation	Selection: After implementing the HHFKA, a significantly greater proportion of students selected fruit (17.8% percentage point increase); *p* < 0.001) and whole grains (67.4% percentage point increase; *p* < 0.001). There was no association with overall vegetable selection. Consumption: The HHFKA was associated with a decrease in milk consumption (61.1% vs. 78.8% consumed; *p* < 0.01). There was no association with total fruit, total vegetable, or whole grain consumption among those who selected the meal component.	Low
Ishdorj et al. 2015 [98]	Texas; 3 elementary schools (students in grades K-5)	QE: Pre/post (no comparison group)	Spring 2012 and Fall 2012	Policy: HHFKA	Aggregate plate waste	Selection: Not measured Consumption: The HHFKA was not associated with differences in consumption of entrées or vegetables.	Very High
Schwartz et al. 2015 [25]	N.S.; 12 middle schools (students in grades 5–7)	QE: Pre/post (no comparison group)	2012, 2013, and 2014	Policy: HHFKA	Weighed plate waste	Selection: The HHFKA was associated with a significant increase in fruit selection. Consumption: The HHFKA was associated with an increase in vegetable and entrée consumption. There was no association with fruit or milk consumption.	Low
Access to Competitive Foods							
Cullen et al. 2000 [125]	TX; 4 elementary schools and 1 middle school (594 students in grade 4–5)	Cross-sectional	1998–1999	Policy: Access to competitive foods	Lunch food records (student self-report)	Selection: Not measured Consumption: Compared with students who had access to competitive foods, students who did not have access consumed significantly more fruits (0.24 vs. 0.11 servings; *p* < 0.001) and vegetables (0.54 vs. 0.47 servings; *p* < 0.05).	High
Cullen et al. 2004 [126]	TX; 4 elementary schools and 1 middle school (594 students in grade 4–5)	QE: Pre/post (with comparison group)	1998–99 to 1999–2000	Policy: Access to competitive foods	Lunch food records (student self-report)	Selection: Not measured Consumption: When students gained access to competitive foods, they consumed on average significantly fewer servings of fruits, vegetables (excluding high-fat vegetables), and milk.	High
Cullen et al. 2006 [129]	Harris County, TX; 3 middle schools (7473 food diaries from students in grades 6–8)	QE: Pre/post (no comparison group)	2001–02 to 2002–03	Policy: Local competitive food policy that included the removal of vending machines from inside cafeteria (moved to hallways near the cafeteria by the gyms) and removal of chips, desserts, and SSBs from snack bars (but still available in vending machines)	Lunch food records (student self-report)	Selection: Not measured Consumption: The policy was associated with a significant increase in milk consumption, and a significant decrease in SSB and vegetable consumption. Compensation was observed between a decrease in a la carte sales from the snack bars and an increase from the vending machines (in their new locations).	High
Cullen et al. 2008 [128]	TX; 3 middle schools (18,178 food diaries from students in grades 6–8)	QE: Pre/post (no comparison group)	2001–02, 2002–03, and 2005–06	Policy: State competitive food policy that restricted the portion size of snacks and SSBs; limited the total fat content of snacks; and limited the frequency of serving high-fat vegetables (i.e., French fries) to ≤3 times per week	Lunch food records (student self-report)	Selection: Not measured Consumption: The policy was associated with greater school meal consumption of vegetables and milk. It was also associated with a decrease in competitive foods (i.e., SSB and snack chips).	High
Ishdorj et al. 2013 [110]	Nationally representative sample (SNDA-III); 256 schools (2096 students)	Cross-sectional	2004–05	(1) Nutrition Education: Availability of nutrition education lessons for every grade in the school (2) Policy: restrictions on competitive foods (e.g., limited à la carte sales, no stores or snack bars selling competitive foods, and fundraisers), French fries, dessert, and whole/ 2% milk; and increases in the amount of fresh F/V available daily at lunch	24 h recall	Selection: Not measured Consumption: Policies that place restrictions on the sales of competitive foods were associated with greater fruit consumption. Policies that restrict desserts were associated with greater vegetable consumption. Policies that limited French fries were associated with lower fruit consumption. Limiting whole and 2% milk was associated with greater fruit and vegetable consumption. Nutrition education and policies that increase the amount of fresh fruit and vegetable available daily at lunch policies were not associated with consumption of fruits or vegetables.	High
Marlette et al. 2005 [127]	Frankfort, KY; 3 middle schools (743 students in grade 6)	Cross-sectional	2002	Policy: Competitive Foods. When competitive foods are available, the impact of purchasing snacks on meal consumption was assessed	Weighed plate waste	Selection: Not measured Consumption: Students who purchased competitive foods consumed on average significantly less fruits, grains, meats, and mixed dished from their school lunch compared with students with only a school lunch.	Low
Other Local Policies							
Canterberry et al. 2017 [133]	New Orleans, LA; 7 elementary schools with 3 food service providers (18,070 trays from students in grades 4 and 5)	QE: Post-only (with comparison group)	2014	Policy: A local policy exceeded the HHFKA and included more fresh, less processed ingredients including: fresh/frozen F/V; more whole grains; no mechanically separated meat or animal by-products; no processed cheese with additives/fillers; and no deep-fried foods	Weighed plate waste	Selection: Not measured Consumption: On average, there were lower school meal consumption rates among the intervention schools, although this was primarily driven by low consumption rates with one of the food service providers. With another food service provider, there were no significant differences in consumption between the intervention schools and control schools.	Low
Cohen et al. 2012 [56]	Boston, MA; 4 middle schools (3049 students in grades 3–8)	QE: Post-only (with comparison group)	2009	(1) Palatability: A professional chef trained cafeteria staff to prepare healthier school lunches (2) Policy: Chocolate milk was removed	Weighed plate waste	Selection: The intervention was associated with a 51% increase in whole grain selection (*p* = 0.02). Consumption: Students in the intervention schools, consumed 0.36 more servings of vegetables per day (*p* = 0.01) compared with students in control schools. There was no impact on milk, fruit, or whole grain consumption.	Low
Cohen et al. 2014 [24]	MA; 4 elementary/K-8 schools (1030 students in grades 3–8)	QE: Pre/post (no comparison group)	Fall 2011 and Fall 2012	Policy: HHFKA + removal of chocolate milk	Weighed plate waste	Selection: The HHFKA was associated with a 23% increase in the percent of students selecting a fruit (*p* < 0.0001). There was no association with vegetable selection. Milk selection decreased by 24.7% (*p* < 0.0001) when chocolate milk was removed. Consumption: The HHFKA was associated with increased entrée consumption (15.6% increase; *p* < 0.0001) and vegetable consumption (16.2% increase; *p* < 0.0001). Milk consumption decreased by 10% when chocolate milk was removed (*p* < 0.0001). There was no impact on fruit consumption.	Low
Farris et al. 2019 [132]	VA; 7 elementary schools (1813 breakfasts from students in grades PK-5)	QE: Pre/post (no comparison group)	2014–15	Policy: Breakfast in the classroom	Visual estimation	Selection: Not measured Consumption: Breakfast in the classroom was associated with decreased overall food waste (43.0% to 38.5%), including decreases for entrée items, juice, and savory snack foods (*p* < 0.01).	High
Hanks et al. 2014 [130]	OR + Midwest and Eastern Regions: 25 elementary schools (students in grades K-5)	QE: Post-only (with comparison group)	2010–11 to 2011–12	Policy: Removal of chocolate milk	Aggregate waste and Visual estimation	Selection: When chocolate milk was removed, 90.1% of sales were replaced with white milk. Consumption: Milk waste was higher in schools that did not have chocolate milk compared with schools that did have chocolate milk.	High
Schwartz et al. 2018 [131]	New England region; 2 K-8 schools (13,883 trays from students in grades K-8)	QE: Pre/post (no comparison group) and Post-only (no comparison group) * *For Policy only	2010–11 to 2012–13	(1) Policy: Removal of chocolate milk (2) Other: Availability of juice	Weighed plate waste	Selection: Significantly fewer students selected milk when juice was available. There was approximately a 20 percentage point increase in milk selection in the second year of the policy compared with the in the first year. Consumption: Among students who selected milk, milk consumption was lower in the second year of the policy compared with in the first year. On days when juice was offered, students consumed significantly less milk (at both time points).	High
Young et al. 2013 [47]	N.S.; 1 middle school (3810 trays from students in grades 6–8)	Cross-Sectional	2011–12	Policy: A new wellness policy required schools to implement the practices below: (1) Choices: Different fruits and vegetables were served each day of the week (2) Nutrition Education: There was an increase in student contact hours for health and physical education	Visual estimation	Selection: Not measured Consumption: After exposure to the wellness policy for over a semester, students consumed significantly more fruits and cooked vegetables.	High

F/V: fruits and vegetables; QE: Quasi-Experimental; RCT: Randomized controlled trial. ^1^ Study designs were defined as: (1) Cross-sectional- Observational study with the exposure and outcome measured simultaneously (no comparison group); (2) Quasi-Experimental (QE): Post Only—Intervention study with a comparison group and data collected post-implementation (no baseline measurements); (3) Quasi-Experimental (QE): Pre/Post—Intervention studies with pre-implementation (i.e., baseline) and post-implementation measurements, with or without a comparison group (non-random allocation of intervention/comparison groups); (4) Randomized Controlled Trial (RCT)—Intervention study with random allocation to intervention or control status and both pre-implementation (baseline) and post-implementation measurements. ^2^ Risk of Bias was based on adapted Newcastle–Ottawa Scales (NOS) for cross-sectional and cohort studies (Supplementary Materials Table S1).

## 4. Discussion

To our knowledge, this is the first systematic review of the literature that comprehensively examined initiatives, interventions, and policies associated with school meal consumption. The factors were at the school meal level (choices, food preparation, and taste tests); the cafeteria environment level (choice architecture, nutrition education, school lunch duration, and recess before lunch); and the policy level (local, state, and federal policies). The findings suggest that several practices are consistently associated with improved meal consumption. These include: (1) offering students choices within each meal component (particularly fruits and vegetables); (2) enhancing the palatability/cultural appropriateness of meals; (3) pre-slicing fruit; (4) incentivizing students to taste fruits and vegetables with rewards; (5) providing more time to eat lunch with longer lunch periods; (6) implementing recess before lunch; and (7) limiting access to competitive foods.

Many of these strategies can be implemented with minimal costs and/or additional labor. First, providing students with 30-min lunch periods can help to ensure that students have enough time to eat after accounting for time spent getting to the cafeteria and waiting in the lunch line. While some schools may be apprehensive to replace academic time with longer lunch durations, in prior research, teachers have reported that students are more attentive in class (and thus more efficient learners) with this policy and that this time in the cafeteria can be valuable for social and emotional learning [134]. Another concern that has been raised regarding longer lunch periods is disruptive student behavior in the cafeteria if they finish their meals quickly; however, implementing recess before lunch has been found to create a calmer lunchroom environment, suggesting that combining these two strategies would be beneficial for consumption and student behavior [134].

Offering more fruit and vegetable choices (especially with pre-sliced fruits such as apples and oranges) was also found to be an effective method to increase consumption. Cost-effective strategies to increase the amount and variety of fruit available in schools include using USDA commodity foods; integrating foods from school gardens; and procuring locally grown produce. The USDA Department of Defense Fresh Fruit and Vegetable Program also provides fresh produce for schools. Restricting access to competitive foods can also be implemented in a cost-neutral manner; prior research has documented that when students have limited access to competitive foods, they instead participate more in the NSLP [135]. These increases in school meal sales have been found to offset revenue losses from the decreased sales of snacks and beverages [135]. While chef-based initiatives to enhance the palatability and cultural appropriateness of meals can be expensive, partnering with volunteer chefs from local restaurants can be a cost-effective solution. Additionally, schools can hire a chef when an existing cafeteria staff member retires, thus enabling this to be a more affordable strategy. Lastly, various free resources are available to schools through the USDA, state departments of educations, and non-profit organizations such as those that provide recipes and culinary workshops.

The findings from this review also highlight some strategies with mixed empirical support. Nutrition education was found to have mixed results when examining its impact on consumption in the cafeteria. This may have occurred due to several reasons. First, the nutrition education curricula across the studies varied in intensity, dose, and content. It is possible that greater time is necessary to dedicate to nutrition education, as well as more staff development and training to have improved effectiveness [136]. Second, it is possible that nutrition education is necessary but not sufficient on its own to reliably influence consumption, especially if there are other obstacles, such as insufficient time to eat. Although it may be possible to improve student intake in the short term without incorporating nutrition education, understanding how our food choices affect our health should still be considered an integral component of the Whole School, Whole Child, Whole Community model and may have several other benefits outside of the lunchroom and over the long term as students become more independent and make their own food choices [136,137].

The Smarter Lunchroom movement has gained traction in the United States with over 29,000 schools implementing these techniques to nudge students towards the healthier options available. Somewhat surprisingly, the results of this systematic review found that some of the commonly cited Smarter Lunchroom techniques had little to no impact on school meal intake [89]. One exception was when students were rewarded with small prizes such as stickers, classroom parties, or t-shirts for eating school meal components. While there may be additional concerns that providing rewards for a preferred behavior may potentially decrease the motivation and/or create negative associations with performing that activity, parenting research more broadly has found inconsistent results [138]. However, this strategy may be burdensome and expensive to implement and maintain. Additionally, the results were mixed when examining the sustained impact on consumption after the interventions concluded. Importantly, the more traditional techniques (e.g., attractive bowls, verbal prompts, creative names, etc.) have generally been found to be effective at improving the selection of certain meal components, especially fruits and vegetables, in prior systematic reviews [29,30,31,32]. Therefore, schools should not abandon these practices, but also not rely only on these approaches to improve students’ school meal intakes. Future studies should examine the combined effect of choice architecture techniques with other potentially successful strategies, such as longer lunch periods, recess before lunch, enhanced palatability/cultural appropriateness of meals, and limited access to competitive foods.

This study has several limitations. First, many studies had a high or very high risk of bias based on NOS scores. Conducting studies with sufficiently large sample sizes, longer periods of observation, obtaining informed consent from students for repeated data collection, and using validated dietary assessment methods can be expensive, highlighting the need for additional resources and grant funding for researchers. It is also noteworthy that when examining the studies with a low risk of bias, the conclusions did not change. Second, publication bias may have been an issue; however, a substantial number of studies examined found no significant associations with school meal intake. Third, the measures of school meal consumption varied between the studies, and some methods may not have been sensitive enough to detect the levels of change often observed in school-based interventions. However, multiple strategies were still found to improve intakes using the various measures. Additionally, while many studies of breakfast in the classroom have been conducted, most were excluded as they were evaluating universal school meals and not the independent impact of breakfast in the classroom. Therefore, future studies should examine this policy to determine whether it has additional benefits beyond universal free breakfast in the cafeteria. Lastly, the studies included in this systematic review were conducted only in the United States. While the findings are likely generalizable to other economically developed countries with strong nutrition standards, future studies should examine these strategies in other locations. A strength of this systematic review was the large number of studies evaluated. In addition, this review began with the conceptual framework outlined in Figure 1 and had strict consumption measurement criteria (described in Table 1).

## 5. Conclusions

Overall, this review suggests that many strategies have the potential to improve school meal consumption. The majority of studies in the current review found improvements in school meal intake when students were provided with multiple choices on the lunch line; pre-sliced fruits; recipes that focused on improving the palatability and cultural appropriateness of the foods offered; longer lunch period; recess before lunch; limiting access to competitive foods; and providing incentives for students to taste the fruits and vegetables offered at lunch. However, commonly used Smarter Lunchroom techniques were not found to be an effective strategy to increase intake of school lunch. While findings were mixed regarding nutrition education’s impact on meal consumption, research has found other benefits of nutrition education to students. Further research is needed regarding school wellness policies and other district-level policies, including limited access to chocolate milk. Importantly, the results suggest that weakening the HHFKA would not be an effective strategy to reduce school meal waste. Instead, school districts and policy makers should consider the multiple strategies found to improve school meal consumption.

## Data Availability

No new data were created or analyzed in this study. Data sharing is not applicable to this article.

## Supplementary Materials

The following are available online at www.mdpi.com/article/10.3390/nu13103520/s1, Table S1: Quality Assessment for Cross-Sectional, Cohort, and Quasi-experimental Studies based on the Newcastle Ottawa Quality Assessment Form.
