# Supplementary material for: Strategies to Improve School Meal Consumption: A Systematic Review
Source: Nutrients. 2021 Oct 7;13(10):3520. doi: 10.3390/nu13103520 (PMC8538164; doi:10.3390/nu13103520)
Supplement: Supplementary file 1 [file nutrients-13-03520-s001.zip › nutrients-1388337-supplementary.pdf]

nutrients MDPI

Supplemental Table S1. Quality Assessment for Cross-Sectional, Cohort, and Quasi-experimental Studies based on the Newcastle Ottawa Quality Assessment Form [1]

	Selection	Comparability		Outcome				
**Author**	Sample size satisfactory[1] (++)	Comparison group[2] (+)	Comparability of subjects in different outcome groups; Confounding factors controlled[3] (++)	Outcome measured at baseline[4] (+)	Assessment of outcome[5] (++)	Statistical test[6] (++)	Total score (max 10)	Risk of bias[7]
**Adams et al. 2005 [2]**	+	+	+		++		5	High
**Adams et al. 2015 [3]**	++	+	++		++	++	9	Low
**Alaimo et al. 2015 [30]**	++	+	+	+	+	++	8	Low
**Amin et al. 2015 [31]**	+			+	++	+	5	High
**Ang et al. 2019 [4]**	++	+	++		+	++	9	Low
**Auld et al. 1998 [32]**	++	+	+	+	+	++	8	Low
**Auld et al. 1999 [33]**	+	+	+	+	+	++	8	Low
**Bates et al. 2015 [34]**	+		+	+	+	++	6	High
**Bean et al. 2018 [35]**	+			+	+	+	4	High
**Bergman et al. 2004 [5]**	+	+			++		4	High
**Bergman et al. 2004 B [6]**	+	+			++		4	High
**Blakeway et al. 1978 [36]**	++	+		+		+	5	High
**Blom-Hoffman et al. 2004 [37]**		+		+	+	+	4	High

	**Selection**	**Comparability**		**Outcome**				
**Author**	Sample size satisfactory[1] (++)	Comparison group[2] (+)	Comparability of subjects in different outcome groups; Confounding factors controlled[3] (++)	Outcome measured at baseline[4] (+)	Assessment of outcome[5] (++)	Statistical test[6] (++)	Total score (max 10)	Risk of bias[7]
**Blondin et al. 2018 [7]**	++	+	+		++	++	8	Low
**Bontranger Yoder et al. 2014 [38]**	++		+	+	+	++	7	Low
**Bontranger Yoder et al. 2015 [8]*** <br>***Exposures excluding "Policy"**	++				+	++	5	High
**Bontranger Yoder et al. 2015 [8]*** <br>***Policy only**	++			+	+	++	6	High
**Burgess-Champoux et al. 2008 [39]**	+	+	+	+	+	+	6	High
**Canterberry et al. 2017 [9]**	+	+	+		++	++	7	Low
**Chapman et al. 2017 [10]**	+	+	+		++	++	7	Low
**Cohen et al. 2012 [12]**	+	+	+		++	++	7	Low
**Cohen et al. 2014 [40]**	++		++	+	++	++	9	Low

	Selection	Comparability		Outcome				
**Author**	Sample size satisfactory[1] (++)	Comparison group[2] (+)	Comparability of subjects in different outcome groups; Confounding factors controlled[3] (++)	Outcome measured at baseline[4] (+)	Assessment of outcome[5] (++)	Statistical test[6] (++)	Total score (max 10)	Risk of bias[7]
**Cohen et al. 2015 [41]**	++	+	++	+	++	++	10	Low
**Cohen et al. 2016 [11]**	++	+	++		++	++	9	Low
**Cohen et al. 2019 [13]**	++	+	++	+	++	++	10	Low
**Cullen et al. 2000 [14]**	+	+	+		+	+	5	High
**Cullen et al. 2004 [42]**	++	+	+	+		+	6	High
**Cullen et al. 2006 [43]**	++		+	+	+	+	6	High
**Cullen et al. 2008 [44]**	++			+	+	+	5	High
**Cullen et al. 2015A [15]**	++	+	+		+	+	6	High
**Cullen et al. 2015B [45]**	++		+	+	+	++	7	Low
**D'Adamo et al. 2021 [46]**	+			+	++	+	5	High
**Elsbernd et al. 2016 [47]**			++	+	+		4	High
**Epstein-Solfield et al. 2018 [48]**	+		++	+	+	+	6	High
**Farris et al. 2019[49]**	++			+	+	+	5	High
**Fenton et al. 2015 [16]**	++	+	++		+	+	7	Low

	**Selection**	**Comparability**		**Outcome**				
**Author**	Sample size satisfactory[1] (++)	Comparison group[2] (+)	Comparability of subjects in different outcome groups; Confounding factors controlled[3] (++)	Outcome measured at baseline[4] (+)	Assessment of outcome[5] (++)	Statistical test[6] (++)	Total score (max 10)	Risk of bias[7]
**Fritts et al. 2019[50]**	+			+	++	+	5	High
**Georgiou (1998 [Gov't Report]) [51]**		+		+	++		4	High
**Getlinger et al. 1996 [52]**				+	++	+	4	High
**Goto et al. 2013 [54]**	+	+		+	++	+	6	High
**Greene et al. 2017 [53]**	++	+	+	+	+	++	8	Low
**Gross et al. 2018 [17]**	++	+	+		+	++	7	Low
**Gustafson et al. 2017 [55]**	+	+	+	+	+	+	6	High
**Hakim et al. 2013 [56]**	+			+	++	+	5	High
**Hamdi et al. 2020 [57]**	++		+	+	++	+	7	Low
**Hanks et al. 2012 [58]**	+			+	++	+	5	High
**Hanks et al. 2013 [59]**	+			+	+	+	4	High
**Hanks et al. 2014 [18]**	++	+				+	4	High
**Head 1974 [60]**	++	+		+	++	+	7	Low
**Hendy et al. 2005 [61]**	+		++	+	+	+	6	High

	**Selection**	**Comparability**		**Outcome**				
**Author**	Sample size satisfactory[1] (++)	Comparison group[2] (+)	Comparability of subjects in different outcome groups; Confounding factors controlled[3] (++)	Outcome measured at baseline[4] (+)	Assessment of outcome[5] (++)	Statistical test[6] (++)	Total score (max 10)	Risk of bias[7]
**Hoffman et al. 2010 [62]**	+	+	++	+	++	+	8	Low
**Hoffman et al. 2011 [63]**	+	+	++	+	++	+	8	Low
**Hudgens et al. 2017 [64]**	+			+	+	+	4	High
**Hunsberger et al. 2014 [19]**	+		+		++		4	High
**Ishdorj et al. 2013 [20]**	++		++		+	+	6	High
**Ishdorj et al. 2015 [65]**	++			+			3	Very High
**Johnson et al. 2017 [21]**	++	+			+	++	6	High
**Jones et al. 2014 [66]**	+			+		+	3	Very High
**Jones et al. 2015 [22]**	+	+			+	++	5	High
**Just et al. 2012 [23]**	++	+	+			++	6	High
**Just et al. 2014 [67]**	+			+	+	+	4	High
**Koch et al. 2020 [68]**	++			+	+	+	5	High
**Larson et al. 2018 [69]**	+	+		+	+	+	5	High
**Liquori et al. 1998 [70]**	+	+		+	+	+	5	High

	**Selection**	**Comparability**		**Outcome**				
**Author**	Sample size satisfactory[1] (++)	Comparison group[2] (+)	Comparability of subjects in different outcome groups; Confounding factors controlled[3] (++)	Outcome measured at baseline[4] (+)	Assessment of outcome[5] (++)	Statistical test[6] (++)	Total score (max 10)	Risk of bias[7]
**Machado et al. 2020 [71]**	+			+	+	+	4	High
**Marlette et al. 2005 [24]**	++		++		++	+	7	Low
**Mazzeo et al. 2017 [72]**	+	+		+	+	+	5	High
**McCool et al. 2005 [73]**				+		+	2	Very High
**McLoughlin et al. 2019 [25]**	+	+	+		++	+	6	High
**Miller et al. 2015 [74]**	+		++	+	++	+	7	Low
**Morrill et al. 2016 [75]**	++	+	++	+	+	++	9	Low
**Perry et al. 2004 [76]**	++	+	++	+	+	++	9	Low
**Prescott et al. 2019 [77]**	+	+	+	+	+	+	6	High
**Price et al. 2015 [78]**	++	+	+	+	+	++	8	Low
**Quinn et al. 2018 [79]**	++			+	+	+	5	High
**Ramsay et al. 2013 [80]**	+		+	+	++	+	6	High
**Redden et al. 2015 [81]**	+			+		+	3	Very High
**Reicks et al. 2012 [82]**	+			+		+	3	Very High

	**Selection**	**Comparability**		**Outcome**				
**Author**	Sample size satisfactory[1] (++)	Comparison group[2] (+)	Comparability of subjects in different outcome groups; Confounding factors controlled[3] (++)	Outcome measured at baseline[4] (+)	Assessment of outcome[5] (++)	Statistical test[6] (++)	Total score (max 10)	Risk of bias[7]
**Reynolds et al. 2000 [83] (*based on the subsample with cafeteria measurements)**	++	+	+	+	+	++	7	Low
**Schwartz 2007 [26]**	+	+			+	+	4	High
**Schwartz et al. 2015 [84]**	++		+	+	++	+	7	Low
**Schwartz et al. 2018 [27]**	+		+	+	++	+	6	High
**Serebrennikov et al. 2020 [85]**	+	+	++	+	+	++	9	Low
**Sharma et al. 2019 [86]**	+	+	++	+	++	+	8	Low
**Smathers et al. 2020 [87]**	+			+		+	3	Very High
**Strohbehn et al. 2016 [88]**	++			+	++	+	6	High
**Swanson et al. 2009 [89]**	+			+	+	+	4	High
**Tanaka et al. 2005 [90]**	+			+		+	3	Very High
**Taylor et al. 2018 [91]**	+	+		+	+	+	5	High
**Thompson et al. 2017 [92]**	+		++	+	++	+	7	Low

	Selection	Comparability		Outcome				
**Author**	Sample size satisfactory[1] (++)	Comparison group[2] (+)	Comparability of subjects in different outcome groups; Confounding factors controlled[3] (++)	Outcome measured at baseline[4] (+)	Assessment of outcome[5] (++)	Statistical test[6] (++)	Total score (max 10)	Risk of bias[7]
**Wansink et al. 2013 [93]**	++	+		+	+	++	7	Low
**Wansink et al. 2015 [94]**	+			+	+	+	4	High
**Wengreen et al. 2013 [95]**	+		++	+	+	+	6	High
**Young et al. 2013 [28]**	+			+	+	+	4	High
**Zellner et al. 2016 [96]**				+	+	+	3	Very High
**Zellner et al. 2017 [29]**		+		+		+	3	Very High

1. **Sample size satisfactory**: Satisfactory simple size (>100 units of analysis (e.g., trays, students, classrooms, schools) AND three or more schools in the intervention condition (++), Satisfactory simple size (>100 units of analysis (e.g., trays, students, classrooms, schools) OR three or more schools in the intervention condition (+), *versus* no information provided or not satisfactory (<100 participants and fewer than three schools in the intervention condition).
2. **Comparison group.** An unexposed group serves as a comparison for the intervention condition (+) *versus* no comparison group.
3. **Comparability of groups; Confounding factors controlled**: Comparability of subjects in different outcome groups and analyses adjusted for relevant predictors/risk factors/confounders, including repeated measures, where appropriate (++), adjusted for some but not all relevant predictors/risk factors/ confounders (+), *versus* information not provided or analyses not adjusted for relevant predictors/risk factors/ confounders.
4. **Outcome measured at baseline**: Baseline measurements collected (+) *versus* no baseline assessments.
5. **Assessment of outcome**: Objective assessment (plate waste) (++), validated non-objective measure (visual estimation; dietary recall) (+), *versus* non-objective and non-validated measure (aggregate plate waste is not a valid approach).
6. **Statistical test**: Statistical tests used to analyze the data clearly described and appropriate, measures of association presented include confidence intervals and/or probability level (p value) AND statistical tests account for clustering of observations, where appropriate (++), Statistical tests used to analyze the data clearly described and appropriate, measures of association presented include confidence intervals and/or probability level (p value), OR statistical tests account for clustering of observations, where appropriate (+), *versus* statistical tests not appropriate, not described, or incomplete.
7. Total score for the Newcastle–Ottawa Scale (NOS) is attributed to a following categories: very high risk of bias (0–3 NOS points), high risk of bias (4–6 NOS points), and low risk of bias (7–10 NOS points)

References

1. Wells, G.A.; Shea, B.; O'Connell, D.a.; Peterson, J.; Welch, V.; Losos, M.; Tugwell, P. The Newcastle-Ottawa Scale (NOS) for assessing the quality of nonrandomised studies in meta-analyses. Oxford: 2000.
2. Adams, M.A.; Pelletier, R.L.; Zive, M.M.; Sallis, J.F. Salad bars and fruit and vegetable consumption in elementary schools: a plate waste study. *Journal of the American Dietetic Association* **2005**, *105*, 1789-1792.
3. Adams, M.A.; Bruening, M.; Ohri-Vachaspati, P.; Hurley, J.C. Location of school lunch salad bars and fruit and vegetable consumption in middle schools: A cross-sectional plate waste study. *Journal of the Academy of Nutrition and Dietetics* **2016**, *116*, 407-416.
4. Ang, I.Y.H.; Wolf, R.L.; Koch, P.A.; Gray, H.L.; Trent, R.; Tipton, E.; Contento, I.R. School lunch environmental factors impacting fruit and vegetable consumption. *Journal of nutrition education and behavior* **2019**, *51*, 68-79.
5. Bergman, E.A.; Buergel, N.S.; Englund, T.F.; Femrite, A. The relationship between the length of the lunch period and nutrient consumption in the elementary school lunch setting. *J Child Nutr Manage* **2004**, *28*.
6. Bergman, E.A.; Buergel, N.S.; Englund, T.F.; Femrite, A. The relationship of meal and recess schedules to plate waste in elementary schools. *J Child Nutr Manag* **2004**, *28*, 1-10.
7. Blondin, S.A.; Goldberg, J.P.; Cash, S.B.; Griffin, T.S.; Economos, C.D. Factors influencing fluid milk waste in a Breakfast in the Classroom School Breakfast Program. *Journal of nutrition education and behavior* **2018**, *50*, 349-356. e341.
8. Yoder, A.B.B.; Foecke, L.L.; Schoeller, D.A. Factors affecting fruit and vegetable school lunch waste in Wisconsin elementary schools participating in Farm to School programmes. *Public health nutrition* **2015**, *18*, 2855-2863.
9. Canterberry, M.; Francois, S.; van Hattum, T.; Rudov, L.; Carton, T.W. School Lunch Consumption Among 3 Food Service Providers in New Orleans. *Journal of School Health* **2018**, *88*, 93-100.
10. Chapman, L.E.; Cohen, J.; Canterberry, M.; Carton, T.W. Factors Associated with School Lunch Consumption: Reverse Recess and School "Brunch". *Journal of the Academy of Nutrition and Dietetics* **2017**, *117*, 1413-1418.
11. Cohen, J.F.; Jahn, J.L.; Richardson, S.; Cluggish, S.A.; Parker, E.; Rimm, E.B. Amount of time to eat lunch is associated with children's selection and consumption of school meal entrée, fruits, vegetables, and milk. *Journal of the Academy of Nutrition and Dietetics* **2016**, *116*, 123-128.
12. Cohen, J.F.; Smit, L.A.; Parker, E.; Austin, S.B.; Frazier, A.L.; Economos, C.D.; Rimm, E.B. Long-term impact of a chef on school lunch consumption: findings from a 2-year pilot study in Boston middle schools. *Journal of the Academy of Nutrition and Dietetics* **2012**, *112*, 927-933.
13. Cohen, J.F.; Richardson, S.; Rimm, E.B. Impact of the Updated USDA School Meal Standards, Chef-Enhanced Meals, and the Removal of Flavored Milk on School Meal Selection and Consumption. *Journal of the Academy of Nutrition and Dietetics* **2019**.
14. Cullen, K.W.; Eagan, J.; Baranowski, T.; Owens, E. Effect of a la carte and snack bar foods at school on children's lunchtime intake of fruits and vegetables. *Journal of the American Dietetic Association* **2000**, *100*, 1482-1486.
15. Cullen, K.W.; Chen, T.-A.; Dave, J.M.; Jensen, H. Differential improvements in student fruit and vegetable selection and consumption in response to the new National School Lunch Program regulations: a pilot study. *Journal of the Academy of Nutrition and Dietetics* **2015**, *115*, 743-750.
16. Fenton, K.; Rosen, N.J.; Wakimoto, P.; Patterson, T.; Goldstein, L.H.; Ritchie, L.D. Eat lunch first or play first? Inconsistent associations with fruit and vegetable consumption in elementary school. *Journal of the Academy of Nutrition and Dietetics* **2015**, *115*, 585-592.
17. Gross, S.M.; Biehl, E.; Marshall, B.; Paige, D.M.; Mmari, K. Role of the elementary school cafeteria environment in fruit, vegetable, and whole-grain consumption by 6-to 8-year-old students. *Journal of nutrition education and behavior* **2019**, *51*, 41-47.
18. Hanks, A.S.; Just, D.R.; Wansink, B. Chocolate milk consequences: a pilot study evaluating the consequences of banning chocolate milk in school cafeterias. *PloS one* **2014**, *9*, e91022.
19. Hunsberger, M.; McGinnis, P.; Smith, J.; Beamer, B.A.; O'Malley, J. Elementary school children's recess schedule and dietary intake at lunch: A community-based participatory research partnership pilot study. *BMC Public Health* **2014**, *14*, 1-7.
20. Ishdorj, A.; Crepinsek, M.K.; Jensen, H.H. Children's consumption of fruits and vegetables: do school environment and policies affect choices at school and away from school? *Applied Economic Perspectives and Policy* **2013**, *35*, 341-359.
21. Johnson, C.C.; Myers, L.; Mundorf, A.R.; O'Malley, K.; Spruance, L.A.; Harris, D.M. Lunch salad bars in New Orleans' middle and high schools: student intake of fruit and vegetables. *International journal of environmental research and public health* **2017**, *14*, 415.
22. Jones, S.J.; Childers, C.; Weaver, A.T.; Ball, J. SC farm-to-school programs encourages children to consume vegetables. *Journal of hunger & environmental nutrition* **2015**, *10*, 511-525.
23. Just, D.R.; Lund, J.; Price, J. The role of variety in increasing the consumption of fruits and vegetables among children. *Agricultural and Resource Economics Review* **2012**, *41*, 72-81.

24. Marlette, M.A.; Templeton, S.B.; Panemangalore, M. Food type, food preparation, and competitive food purchases impact school lunch plate waste by sixth-grade students. *Journal of the American Dietetic Association* **2005**, *105*, 1779-1782.
25. McLoughlin, G.M.; Edwards, C.G.; Jones, A.; Chojnacki, M.R.; Baumgartner, N.W.; Walk, A.D.; Woods, A.M.; Graber, K.C.; Khan, N.A. School Lunch Timing and Children's Physical Activity During Recess: An Exploratory Study. *Journal of nutrition education and behavior* **2019**, *51*, 616-622.
26. Schwartz, M.B. The influence of a verbal prompt on school lunch fruit consumption: a pilot study. *International Journal of Behavioral Nutrition and Physical Activity* **2007**, *4*, 1-5.
27. Schwartz, M.B.; Henderson, K.E.; Read, M.; Cornelius, T. Student acceptance of plain milk increases significantly 2 years after flavored milk is removed from school cafeterias: an observational study. *Journal of the Academy of Nutrition and Dietetics* **2018**, *118*, 857-864.
28. Young, K.D.; Snelling, A.; Maroto, M.; Young, K.A. Consumption of fruits and vegetables in middle school students following the implementation of a school district wellness policy. *The Journal of Child Nutrition and Management* **2013**, *37*, 1-6.
29. Zellner, D.A.; Cobuzzi, J.L. Eat your veggies: A chef-prepared, family style school lunch increases vegetable liking and consumption in elementary school students. *Food Quality and Preference* **2017**, *55*, 8-15.
30. Alaimo, K.; Carlson, J.J.; Pfeiffer, K.A.; Eisenmann, J.C.; Paek, H.-J.; Betz, H.H.; Thompson, T.; Wen, Y.; Norman, G.J. Project FIT: a school, community and social marketing intervention improves healthy eating among low-income elementary school children. *Journal of community health* **2015**, *40*, 815-826.
31. Amin, S.A.; Yon, B.A.; Taylor, J.C.; Johnson, R.K. Impact of the National School Lunch Program on fruit and vegetable selection in northeastern elementary schoolchildren, 2012–2013. *Public Health Reports* **2015**, *130*, 453-457.
32. Auld, G.W.; Romaniello, C.; Heimendinger, J.; Hambidge, C.; Hambidge, M. Outcomes from a school-based nutrition education program using resource teachers and cross-disciplinary models. *Journal of Nutrition Education* **1998**, *30*, 268-280.
33. Auld, G.W.; Romaniello, C.; Heimendinger, J.; Hambidge, C.; Hambidge, M. Outcomes from a school-based nutrition education program alternating special resource teachers and classroom teachers. *Journal of School Health* **1999**, *69*, 403-408.
34. Bates, D.; Price, J. Impact of fruit smoothies on adolescent fruit consumption at school. *Health Education & Behavior* **2015**, *42*, 487-492.
35. Bean, M.K.; Spalding, B.B.; Theriault, E.; Dransfield, K.-B.; Sova, A.; Stewart, M.D. Salad bars increased selection and decreased consumption of fruits and vegetables 1 month after installation in Title I elementary schools: a plate waste study. *Journal of nutrition education and behavior* **2018**, *50*, 589-597.
36. Blakeway, S.F.; Knickrehm, M.E. Nutrition education in the Little Rock school lunch program. *Journal of the American Dietetic Association* **1978**, *72*, 389-391.
37. Blom-Hoffman, J.; Kelleher, C.; Power, T.J.; Leff, S.S. Promoting healthy food consumption among young children: Evaluation of a multi-component nutrition education program. *Journal of School Psychology* **2004**, *42*, 45-60.
38. Yoder, A.B.B.; Liebhart, J.L.; McCarty, D.J.; Meinen, A.; Schoeller, D.; Vargas, C.; LaRowe, T. Farm to elementary school programming increases access to fruits and vegetables and increases their consumption among those with low intake. *Journal of Nutrition Education and Behavior* **2014**, *46*, 341-349.
39. Burgess-Champoux, T.L.; Chan, H.W.; Rosen, R.; Marquart, L.; Reicks, M. Healthy whole-grain choices for children and parents: a multi-component school-based pilot intervention. *Public Health Nutrition* **2008**, *11*, 849-859.
40. Cohen, J.F.; Richardson, S.; Parker, E.; Catalano, P.J.; Rimm, E.B. Impact of the new US Department of Agriculture school meal standards on food selection, consumption, and waste. *American Journal of Preventive Medicine* **2014**, *46*, 388-394.
41. Cohen, J.F.; Richardson, S.A.; Cluggish, S.A.; Parker, E.; Catalano, P.J.; Rimm, E.B. Effects of choice architecture and chef-enhanced meals on the selection and consumption of healthier school foods: a randomized clinical trial. *JAMA Pediatrics* **2015**, *169*, 431-437.
42. Cullen, K.W.; Zakeri, I. Fruits, vegetables, milk, and sweetened beverages consumption and access to a la carte/snack bar meals at school. *American Journal of Public Health* **2004**, *94*, 463-467.
43. Cullen, K.W.; Watson, K.; Zakeri, I.; Ralston, K. Exploring changes in middle-school student lunch consumption after local school food service policy modifications. *Public health nutrition* **2006**, *9*, 814-820.
44. Cullen, K.W.; Watson, K.; Zakeri, I. Improvements in middle school student dietary intake after implementation of the Texas Public School Nutrition Policy. *American Journal of Public Health* **2008**, *98*, 111-117.
45. Cullen, K.W.; Chen, T.-A.; Dave, J.M. Changes in foods selected and consumed after implementation of the new National School Lunch Program meal patterns in southeast Texas. *Preventive Medicine Reports* **2015**, *2*, 440-443.
46. D'Adamo, C.R.; Parker, E.A.; McArdle, P.F.; Trilling, A.; Bowden, B.; Bahr-Robertson, M.K.; Keller, K.L.; Berman, B.M. The addition of spices and herbs to vegetables in the National School Lunch Program increased vegetable intake at an urban, economically-underserved, and predominantly African-American high school. *Food Quality and Preference* **2021**, *88*, 104076.
47. Elsbernd, S.; Reicks, M.M.; Mann, T.L.; Redden, J.P.; Mykerezi, E.; Vickers, Z.M. Serving vegetables first: A strategy to increase vegetable consumption in elementary school cafeterias. *Appetite* **2016**, *96*, 111-115.

48. Epstein-Solfield, A.; Arango, C.; Ogan, D.; Stendell-Hollis, N. The effects of a nutrition education intervention on third-and fifth grade students' fruit and vegetable knowledge, preference and consumption. *Journal of Child Nutrition and Management* **2018**, *42*.
49. Farris, A.R.; Roy, M.; Serrano, E.L.; Misyak, S. Impact of breakfast in the classroom on participation and food waste. *Journal of nutrition education and behavior* **2019**, *51*, 893-898.
50. Fritts, J.R.; Bermudez, M.A.; Hargrove, R.L.; Alla, L.; Fort, C.; Liang, Q.; Cravener, T.L.; Rolls, B.J.; D'Adamo, C.R.; Hayes, J.E. Using Herbs and Spices to Increase Vegetable Intake Among Rural Adolescents. *Journal of nutrition education and behavior* **2019**, *51*, 806-816. e801.
51. Georgiou, C. The Effect of Nutrition Education on Third Graders' School Lunch Consumption in a School Offering Food Pyramid Choice Menus. *Oregon: Oregon State Department of Education, Salem. Child Nutrition Division* **1998**.
52. Getlinger, M.J.; Laughlin, C.V.; Bell, E.; Akre, C.; Arjmandi, B.H. Food waste is reduced when elementary-school children have recess before lunch. *Journal of the Academy of Nutrition and Dietetics* **1996**, *96*, 906.
53. Greene, K.N.; Gabrielyan, G.; Just, D.R.; Wansink, B. Fruit-promoting smarter lunchrooms interventions: results from a cluster RCT. *American Journal of Preventive Medicine* **2017**, *52*, 451-458.
54. Goto, K.; Waite, A.; Wolff, C.; Chan, K.; Giovanni, M. Do environmental interventions impact elementary school students' lunchtime milk selection? *Applied Economic Perspectives and Policy* **2013**, *35*, 360-376.
55. Gustafson, C.R.; Abbey, B.M.; Heelan, K.A. Impact of schoolchildren's involvement in the design process on the effectiveness of healthy food promotion materials. *Preventive medicine reports* **2017**, *6*, 246-250.
56. Hakim, S.M.; Meissen, G. Increasing consumption of fruits and vegetables in the school cafeteria: the influence of active choice. *Journal of health care for the poor and underserved* **2013**, *24*, 145-157.
57. Hamdi, N.; Ellison, B.; McCaffrey, J.; Metcalfe, J.J.; Hoffman, A.; Haywood, P.; Prescott, M.P. Implementation of a Multi-Component School Lunch Environmental Change Intervention to Improve Child Fruit and Vegetable Intake: A Mixed-Methods Study. *International Journal of Environmental Research and Public Health* **2020**, *17*, 3971.
58. Hanks, A.S.; Just, D.R.; Smith, L.E.; Wansink, B. Healthy convenience: nudging students toward healthier choices in the lunchroom. *Journal of public health* **2012**, *34*, 370-376.
59. Hanks, A.S.; Just, D.R.; Wansink, B. Smarter lunchrooms can address new school lunchroom guidelines and childhood obesity. *The Journal of pediatrics* **2013**, *162*, 867-869.
60. Head, M.K. A nutrition education program at three grade levels. *Journal of Nutrition Education* **1974**, *6*, 56-59.
61. Hendy, H.M.; Williams, K.E.; Camise, T.S. "Kids Choice" school lunch program increases children's fruit and vegetable acceptance. *Appetite* **2005**, *45*, 250-263.
62. Hoffman, J.A.; Franko, D.L.; Thompson, D.R.; Power, T.J.; Stallings, V.A. Longitudinal behavioral effects of a school-based fruit and vegetable promotion program. *Journal of pediatric psychology* **2010**, *35*, 61-71.
63. Hoffman, J.A.; Thompson, D.R.; Franko, D.L.; Power, T.J.; Leff, S.S.; Stallings, V.A. Decaying behavioral effects in a randomized, multi-year fruit and vegetable intake intervention. *Preventive medicine* **2011**, *52*, 370-375.
64. Hudgens, M.E.; Barnes, A.S.; Lockhart, M.K.; Ellsworth, S.C.; Beckford, M.; Siegel, R.M. Small prizes improve food selection in a school cafeteria without increasing waste. *Clinical pediatrics* **2017**, *56*, 123-126.
65. Ishdorj, A.; Capps Jr, O.; Storey, M.; Murano, P.S. *Investigating the relationship between food pairings and plate waste from elementary school lunches*; 2015.
66. Jones, B.A.; Madden, G.J.; Wengreen, H.J. The FIT Game: preliminary evaluation of a gamification approach to increasing fruit and vegetable consumption in school. *Preventive medicine* **2014**, *68*, 76-79.
67. Just, D.R.; Wansink, B.; Hanks, A.S. Chefs move to schools. A pilot examination of how chef-created dishes can increase school lunch participation and fruit and vegetable intake. *Appetite* **2014**, *83*, 242-247.
68. Koch, P.A.; Wolf, R.L.; Trent, R.; Guerra, L.A. School Transformation after Redesign of 3 Cafeterias (STARCafé). *Health Behavior and Policy Review* **2020**, *7*, 329-341.
69. Larson, J.N.; Brusseau, T.A.; Wengreen, H.; Fairclough, S.J.; Newton, M.M.; Hannon, J.C. Fit "N" Cool Kids: The Effects of Character Modeling and Goal Setting on Children's Physical Activity and Fruit and Vegetable Consumption. *Clinical Medicine Insights: Pediatrics* **2018**, *12*, 1179556518784296.
70. Liquori, T.; Koch, P.D.; Contento, I.R.; Castle, J. The cookshop program: outcome evaluation of a nutrition education program linking lunchroom food experiences with classroom cooking experiences. *Journal of Nutrition Education* **1998**, *30*, 302-313.
71. Machado, S.S.; Burton, M.; Loy, W.; Chapman, K.A. Promoting school lunch fruit and vegetable intake through role modeling: a pilot study. *AIMS public health* **2020**, *7*, 10.
72. Mazzeo, S.E.; Bean, M.K.; Palmberg, A.A.; Simpson, C.C.; Thacker, L.R.; Dunne Stewart, M.; Gow, R.W. A pilot intervention targeting dietary intake in school cafeterias. *Health Behavior and Policy Review* **2017**, *4*, 256-264.
73. McCool, A.C.; Myung, E.; Chien, T.-C. Modification of the form in which fresh fruit is served as a possible means of increasing the consumption of fruit offered to elementary and middle school students. *Journal of Foodservice Business Research* **2006**, *8*, 73-85.
74. Miller, N.; Reicks, M.; Redden, J.P.; Mann, T.; Mykerezi, E.; Vickers, Z. Increasing portion sizes of fruits and vegetables in an elementary school lunch program can increase fruit and vegetable consumption. *Appetite* **2015**, *91*, 426-430.

75. Morrill, B.A.; Madden, G.J.; Wengreen, H.J.; Fargo, J.D.; Aguilar, S.S. A randomized controlled trial of the Food Dudes program: tangible rewards are more effective than social rewards for increasing short-and long-term fruit and vegetable consumption. *Journal of the Academy of Nutrition and Dietetics* **2016**, *116*, 618-629.
76. Perry, C.L.; Bishop, D.B.; Taylor, G.L.; Davis, M.; Story, M.; Gray, C.; Bishop, S.C.; Mays, R.A.W.; Lytle, L.A.; Harnack, L. A randomized school trial of environmental strategies to encourage fruit and vegetable consumption among children. *Health education & behavior* **2004**, *31*, 65-76.
77. Prescott, M.P.; Burg, X.; Metcalfe, J.J.; Lipka, A.E.; Herritt, C.; Cunningham-Sabo, L. Healthy planet, healthy youth: A food systems education and promotion intervention to improve adolescent diet quality and reduce food waste. *Nutrients* **2019**, *11*, 1869.
78. Price, J.; Just, D.R. Lunch, recess and nutrition: Responding to time incentives in the cafeteria. *Preventive Medicine* **2015**, *71*, 27-30.
79. Quinn, E.L.; Johnson, D.B.; Podrabsky, M.; Saelens, B.E.; Bignell, W.; Krieger, J. Peer reviewed: effects of a behavioral economics intervention on food choice and food consumption in middle-school and high-school cafeterias. *Preventing chronic disease* **2018**, *15*.
80. Ramsay, S.; Safaii, S.; Croschere, T.; Branen, L.J.; Wiest, M. Kindergarteners' entrée intake increases when served a larger entrée portion in school lunch: a quasi-experiment. *Journal of school health* **2013**, *83*, 239-242.
81. Redden, J.P.; Mann, T.; Vickers, Z.; Mykerezi, E.; Reicks, M.; Elsbernd, S. Serving first in isolation increases vegetable intake among elementary schoolchildren. *PloS one* **2015**, *10*, e0121283.
82. Reicks, M.; Redden, J.P.; Mann, T.; Mykerezi, E.; Vickers, Z. Photographs in lunch tray compartments and vegetable consumption among children in elementary school cafeterias. *Jama* **2012**, *307*, 784-785.
83. Reynolds, K.D.; Franklin, F.A.; Binkley, D.; Raczynski, J.M.; Harrington, K.F.; Kirk, K.A.; Person, S. Increasing the fruit and vegetable consumption of fourth-graders: results from the high 5 project. *Preventive medicine* **2000**, *30*, 309-319.
84. Schwartz, M.B.; Henderson, K.E.; Read, M.; Danna, N.; Ickovics, J.R. New school meal regulations increase fruit consumption and do not increase total plate waste. *Childhood Obesity* **2015**, *11*, 242-247.
85. Serebrennikov, D.; Katare, B.; Kirkham, L.; Schmitt, S. Effect of classroom intervention on student food selection and plate waste: Evidence from a randomized control trial. *PloS one* **2020**, *15*, e0226181.
86. Sharma, S.; Marshall, A.; Chow, J.; Ranjit, N.; Bounds, G.; Hearne, K.; Cramer, N.; Oceguera, A.; Farhat, A.; Markham, C. Impact of a pilot school-based nutrition intervention on fruit and vegetable waste at school lunches. *Journal of nutrition education and behavior* **2019**, *51*, 1202-1210. e1201.
87. Smathers, C.A.; Graffagnino, C. Offering Self-Serve Sliced Apples in Bulk Increases Students' Consumption and Decreases Apple Waste in Elementary School Cafeterias. *Journal of Child Nutrition & Management* **2020**, *44*, n1.
88. Strohbehn, C.H.; Strohbehn, G.W.; Lanningham-Foster, L.; Litchfield, R.E.; Scheidel, C.; Delger, P. Impacts of scheduling recess before lunch in elementary schools: A case study approach of plate waste and perceived behaviors. *The Journal of Child Nutrition and Management* **2016**, *40*.
89. Swanson, M.; Branscum, A.; Nakayima, P.J. Promoting consumption of fruit in elementary school cafeterias. The effects of slicing apples and oranges. *Appetite* **2009**, *53*, 264-267.
90. Tanaka, C.; Richards, K.L.; Takeuchi, L.S.; Otani, M.; Maddock, J. Modifying the recess before lunch program. *Californian Journal of Health Promotion* **2005**, *3*, 1-7.
91. Taylor, J.C.; Zidenberg-Cherr, S.; Linnell, J.D.; Feenstra, G.; Scherr, R.E. Impact of a multicomponent, school-based nutrition intervention on students’ lunchtime fruit and vegetable availability and intake: a pilot study evaluating the Shaping Healthy Choices Program. *Journal of Hunger & Environmental Nutrition* **2018**, *13*, 415-428.
92. Thompson, E.; Johnson, D.C.; Leite-Bennett, A.; Ding, Y.; Mehrotra, K. The impact of multiple strategies to encourage fruit and vegetable consumption during school lunch. *Journal of School Health* **2017**, *87*, 616-622.
93. Wansink, B.; Just, D.R.; Hanks, A.S.; Smith, L.E. Pre-sliced fruit in school cafeterias: children's selection and intake. *American journal of preventive medicine* **2013**, *44*, 477-480.
94. Wansink, B.; Hanks, A.S.; Just, D.R. A plant to plate pilot: a cold-climate high school garden increased vegetable selection but also waste. *Acta Paediatrica* **2015**, *104*, 823-826.
95. Wengreen, H.J.; Madden, G.J.; Aguilar, S.S.; Smits, R.R.; Jones, B.A. Incentivizing children's fruit and vegetable consumption: results of a United States pilot study of the Food Dudes Program. *Journal of nutrition education and behavior* **2013**, *45*, 54-59.
96. Zellner, D.A.; Cobuzzi, J.L. Just dessert: Serving fruit as a separate “dessert” course increases vegetable consumption in a school lunch. *Food Quality and Preference* **2016**, *48*, 195-198.
